# Force per cross-sectional area from molecules to muscles: a general property of biological motors

**DOI:** 10.1098/rsos.160313

**Published:** 2016-07-20

**Authors:** Jean-Pierre Rospars, Nicole Meyer-Vernet

**Affiliations:** 1 Institut National de la Recherche Agronomique (INRA), Unité Mixte de Recherche 1392 Institut d'Ecologie et des Sciences de l'Environnement de Paris, 78000 Versailles, France; 2 LESIA, Observatoire de Paris, CNRS, PSL Research University, UPMC, Sorbonne University, Paris Diderot, Sorbonne Paris Cité, 92195 Cedex Meudon, France

**Keywords:** biological motors, specific tension, molecular motors, myofibrils, muscles

## Abstract

We propose to formally extend the notion of specific tension, i.e. force per cross-sectional area—classically used for muscles, to quantify forces in molecular motors exerting various biological functions. In doing so, we review and compare the maximum tensions exerted by about 265 biological motors operated by about 150 species of different taxonomic groups. The motors considered range from single molecules and motile appendages of microorganisms to whole muscles of large animals. We show that specific tensions exerted by molecular and non-molecular motors follow similar statistical distributions, with in particular, similar medians and (logarithmic) means. Over the 10^19^ mass (*M*) range of the cell or body from which the motors are extracted, their specific tensions vary as *M^α^* with *α* not significantly different from zero. The typical specific tension found in most motors is about 200 kPa, which generalizes to individual molecular motors and microorganisms a classical property of macroscopic muscles. We propose a basic order-of-magnitude interpretation of this result.

## 1. Background

Living organisms use biological motors for various functions, which range from internal transport of ions and molecules in cells to motion of microorganisms and animals, the latter being driven by muscles. The forces developed by muscles are generally expressed as force per cross-sectional area, called specific tension or stress. It has been known for a long time that the vertebrate striated muscles can exert maximum tensions at constant length (isometric tension) of about 200–300 kPa which are on first approximation independent of the muscle and the body mass [1]. This rule was extended to arthropod muscles with values in the range 300–700 kPa [2], although in some mollusc muscles stresses up to 1400 kPa were reported [3]. Later, a review of the literature based on muscles of 72 species of different taxonomic groups, including mammals, birds, reptiles, amphibians, molluscs, insects and crustaceans [4] concluded that there was no significant relationship between body mass and isometric tension, although isometric tension was found to be significantly higher in molluscs, crustaceans and amphibians than in other groups.

In the last 20 years, investigations were extended at the subcellular and molecular levels to investigate myofibrils (e.g. [5]), and non-muscular motors (e.g. [6]). The latter included measurement of forces developed by rotary or linear motors operating the *F*_0_*F*_1_-ATPase ion pump (e.g. [7,8]), bacterial flagella (e.g. [9]), bacterial pili (e.g. [10,11]), and the helical spasmoneme spring of the protozoan *Vorticella* (e.g. [12]). Investigations also included forces generated by single molecules producing tension used for locomotion or for other functions. The former include myosin II—a major component of myofibrils driving skeletal muscles (e.g. [13]), and axonemal dynein—bending flagella of eukaryotic cells (e.g. [14]). The latter include conventional kinesin (e.g. [15]), cytoplasmic dynein—transporting various cargos in cells (e.g. [16]), and RNA polymerase—moving along DNA while carrying transcription [17].

Despite their diversity, all these motors are based on protein machines generating forces. Macroscopic muscles are based on the myosin motor, whereas microorganisms and cells use other types of molecular motors. For comparing motors of so many different sizes, the convenient parameter is not the force *F*, which varies from several 10^−12^ N for the myosin globular motor of cross-sectional area *A* ∼ 40 nm^2^ to approximately 500 N for a large muscle of cross section approximately 20 cm^2^, but, as we intend to show, the specific tension *F*/*A* (all symbols and abbreviations are defined in table 1). In muscles, the approximate conservation of *F*/*A* between animals is an extension of a rule dating back to Galileo, that the strength of a structure is proportional to its cross section. Now, it turns out from the above numbers that the tension of the myosin molecular motor is of the same order of magnitude as the tension of macroscopic muscles (all references to tension here and elsewhere refer to specific tension unless otherwise noted). We will show that this property is not a coincidence but stems from the basic arrangement of cross-bridges in striated muscles. Furthermore, because biological molecular motors are based on protein machines that convert chemical energy into mechanical energy in similar ways (with the possible exception of pili and jump muscles), their tensions are expected to be of the same order of magnitude as that of myosin. Therefore, we propose to extend to molecular motors the concept of tension of macroscopic muscles and to compare their applied forces per unit cross-sectional area. That the forces per unit cross-sectional area may be similar for molecular motors and muscles agrees with results by Marden & Allen [18] and Marden [19], who show in a class of motors that maximum force output scales as the two-thirds power of motor's mass, close to the motor's cross-sectional area.

**Table 1.** List of abbreviations

*A*	cross-sectional area of motors
*F*	force exerted by motors
*V*	volume of molecular motors
Al	algae
Am	amphibian
Ar	arachnids
Ba	bacteria
Bi	birds
Cr	crustaceans
DA	axonemal dynein
DC	cytoplasmic dynein
Ec	echinoderms
*f*	specific tension of motors
FA	*F*_0_/*F*_1_ ATPase
FI	muscular fibre
Fi	fishes
FL	flagellum
Fly	fly locomotors
Fu	fungi
In	insects
IQR	interquartile range
KI	kinesin
*m*	mass of molecular motors
*M*	mass of organisms
M1	single molecule
M2	molecular assembly
Ma	mammals
MF	myofibril
Mo	molluscs
MU	muscle *in vitro*
MV	muscle *in vivo*
MY	myosin
non-loc	non-locomotory
PI	pili
Pr	protozoa
Re	reptiles
RN	RNA polymerase
SP	spasmoneme
Swim	swim locomotors
Terr	terrestrial locomotors

In order to make a meaningful comparison, we need to consider a representative set of muscle tensions, as well as the tension of the myosin motor and those of various other molecular motors. So, we analysed 329 published values of maximum forces or tension for approximately 265 diverse biological motors. These motors include single molecules, molecular assemblies, muscle cells and whole muscles with various functional demands. They come from free-living cells and multicellular organisms of diverse phyla spanning more than 18 orders of magnitude in mass from 10^−16^ to 10^3^ kg. Our primary interest was for motors involved in whole body motion, whereas the other motors were kept for comparison.

The three main questions we addressed on this basis are as follows. Can the notion of specific tension of muscles (force per cross-sectional area) be formally extended to propulsion of organelles and to individual molecular motors? How does this tension compare with that in muscles, and can the results be understood in terms of the basic structures of both molecular motors and muscle fibres? How does tension in motors devoted to cell or body motion compare with tension in other motors?

## 2. Material and methods

### 2.1. Motor forces

The main variable of interest in this paper is the force generated by molecules, molecular assemblies, muscle fibres and muscles. Our dataset includes 13 motor types aggregated in five motor classes depending on the nature of the generated force.

(i) Forces generated by single molecules (denoted M1): myosin II, kinesin I, axonemal and cytoplasmic dynein, and RNA polymerase (other classes of myosin and kinesin were not considered because of insufficient data);
(ii) forces produced by large molecular assemblies (denoted M2): *F*_0_*F*_1_-ATPase, bacterial flagella, pili, spasmonemes and myofibrils. These motors can be also classified as non-locomotory (ATPase) and locomotory (the others) or as rotary (ATPase, bacterial flagella) and linear (the others);
(iii) forces produced by single muscle fibres (i. e. muscle cells) or bundles of a few muscle fibres (both denoted FI), frequently demembranated (skinned), while maximally stimulated and clamped at constant length (isometric contraction), with electrical or chemical stimulations;
(iv) maximum force produced by dissected large bundles of fibres or isolated whole muscles stimulated isometrically with electrical stimulation of the nerve or the muscle (denoted MU); and
(v) forces measured in behaving animals engaged in a wide range of activities including running, jumping, swimming and biting (denoted MV).

Single molecules (M1) and molecular assemblies (M2) are collectively called here ‘molecular motors’. The other motors, muscle fibres (FI) and whole muscles (MU and MV) are called ‘non-molecular motors’.

### 2.2. Identification of study reports

Values of forces generated by molecular and non-molecular motors were taken from 173 articles published in peer-reviewed journals for a wide variety of cells and animals. We sought a sample that is representative of the widest range of sizes and design varieties for as many species as possible (approx. 150 species were found) representing several different taxonomic groups, including bacteria, protozoa, algae, fungi, echinoderms, insects, crustaceans, molluscs, fishes, amphibian, reptiles, birds and mammals.

For molecular motors, we searched for articles providing the main variables of interest (either force for linear motors or torque and lever arm for rotary motors) for the 10 types listed above. Other types were not considered. For example, of the 14 classes of kinesin, only the most studied kinesin I was included and in the myosin superfamily which consists of at least 18 classes of motor proteins involved in a large variety of physiological processes, only class II myosin (conventional) responsible for muscle contraction was included; the other classes involved in phagocytosis, cell motility and vesicle transport were excluded. For each type, potentially relevant papers were searched using the Google Scholar database using as keywords the motor type plus ‘force’, ‘torque’ or ‘pN’.

For non-molecular motors, we proceeded in two steps. First, relevant papers were identified from previous review papers [1,2,4,18]; all their cited references were included, except the rare cases for which the full text was not available or the paper could not be feasibly translated into English. Second, other potentially relevant papers were searched without restriction on language or date in the Google Scholar database using keywords (‘specific tension’, ‘muscle stress’, ‘fibre’, ‘fiber’, ‘N/m^2^’, ‘N m^−2^’, ‘N/cm^2^’, ‘N cm^−2^’, ‘N mm^−2^’, ‘pascal’, ‘kPa’, ‘physiological cross-sectional area’, ‘PCSA’, ‘CSA’, etc.). Bibliographic searches were discontinued in April 2015.

The papers in this preliminary list were screened based on their title and abstract to exclude those unrelated to biological motors, then collected. The useful information was extracted from each of them (see below) with independent checks by the two authors for most of them. Papers without original measurements were excluded. Data published more than once by the same author(s) or reproduced by other authors were identified and only the paper with the original measurement was kept in the reference list. Measurements not fulfilling our criteria (stall force of single molecular motor, maximum isometric tension of non-molecular motors) were not considered. No relevant papers were excluded.

### 2.3. Motor tensions

For all motors, the measured forces *F* were normalized per cross-sectional area *A* (tension *f* = *F*/*A* expressed in Newton per square-metre or equivalently kilopascal).

For molecular motors the tensions were calculated from the published values (measured force or for rotary motors, torque and lever arm, tables 2 and 3) with the area *A* calculated from the volume *V* of the motor (with the order-of-magnitude approximation *A* *=* *V*^2/3^, table 2), except for a few elongated shapes (pilus and spasmoneme) for which we estimated *A* from the diameter of the molecular assembly. For myosin, *A* was estimated from the head of the molecule.

**Table 2.** Characteristic sizes of linear and rotary molecular motors. (Abb, abbreviation; *m*, motor mass (in kDa), *m*_pg_ = *αm*_kDa_, with *α* = 10^15^/*N*_A_ pg kDa^−1^, *N*_A_, Avogadro's number; *V*, motor volume (in nm^3^), *V* = *αm*_kDa_/*ρ*, with *ρ* = 10^−9^ pg nm^−3^; *A*, motor cross-section (in nm^2^), *A* = *V*^2/3^; *L*, lever arm (in nm).)

type	motor	Abb	*m* (kDa)	*V* (nm^3^)	*A* (nm^2^)	*L* (nm)	reference
linear	RNA polymerase	RN	590	980	99	—	Mooney and Landick [20]
	dynein (motor part)	DA/DC	331	550	67	—	Reck-Peterson *et al*. [21], Carter *et al*. [22]
	kinesin	KI	120	199	34	—	Block [23]
	myosin	MY	130	216	36	—	Rayment *et al*. [24], Rayment & Holden [25], Goldman [26], Billington *et al*. [27]
rotary	bacterial *F*_0_ ATP synthase	FA	180	299	45	3.5	Yoshida *et al*. [28], Hoffmann *et al*. [29]
	bacterial *F*_1_ ATP synthase	FA	380	631	74	4.5	Yoshida *et al*. [28], Hoffmann *et al*. [29]
	bacterial flagellum	FL	10^4^	1.67 × 10^4^	650	20	Berg [9], Reid *et al*. [30], Minamino *et al*. [31]

**Table 3.** Molecular motors. (No, line number; Ab, abbreviated motor name; Ty, motor type: M1 = single molecule, M2 = molecular assembly, including myofibrils and myocytes; U, organism: U = unicellular, Z = multicellular; C, S = swimming; T = terrestrial, solid surface; F = flying; N = non-locomotory; group, taxonomic group, see list of abbreviations; motor: m. = muscle; *M*, cell or body mass (kg); *I*, mass indicated in the cited article : Y = Yes, N = No; *A*, molecular area (nm^2^); *F*, force (pN) or torque (pN nm)/lever arm (nm) of rotary motors; *f*, specific tension (kPa); T, temperature (°C), R = room temperature; Comment, f. = force.)

no.	Ab	Ty	U	C	species	group	motor	*M* (kg)	*I*	*A* (nm^2^)	*F* (pN)	*f* (kPa)	*T* (°C)	comment	reference
linear motors															
1	RN	M1	U	N	*Escherichia coli*	Ba	RNA polymerase	1.3 × 10^−15^	N	99	25	253	—	stall force	Wang *et al*. [17]
2	DC	M1	U	N	*Saccharomyces cerevisiae* (yeast)	Fu	dynein (cytoplasmic)	3 × 10^−13^	N	67	7	104	25	stall force	Gennerich *et al*. [16]
3	DC	M1	Z	N	*Drosophila melanogaster* (fruit fly)	In	dynein (cytoplasmic, early embryo)	0.9 × 10^−13^	N	67	1.10	16	—	estimate per single dynein	Gross *et al*. [32]
4	DC	M1	Z	N	*Sus scrofa domesticus* (pig)	Ma	dynein (cytoplasmic, brain)	1.6 × 10^−13^	N	67	7.50	112	25	active dynein stall force	Toba *et al*. [33]
5	DC	M1	Z	N	*Bos taurus* (bull)	Ma	dynein (cytoplasmic, brain)	10^−13^	N	67	1.10	16	24	stall force	Mallik *et al*. [34]
6	DA	M1	Z	S	*Tetrahymena thermophile*	Pr	dynein (axonemal, cilia)	3 × 10^−11^	N	67	4.70	70	26	single molecule	Hirakawa *et al*. [35]
7	DA	M1	Z	S	*Chlamydomonas reinhardtii*	Al	dynein (axonemal, flagellum)	5 × 10^−13^	N	67	1.20	18	—	trap force	Sakakibara *et al*. [36]
8	DA	M1	U	S	*Hemicentrotus pulcherrimus*	Ec	dynein (axonemal, sperm)	10^−13^	N	67	6	90	25	isolated arms	Shingyoji *et al*. [37]
9	DA	M1	U	S	*Bos taurus* (bull)	Ma	dynein (axonemal, flagellum sperm)	10^−13^	N	67	5	75	—	isometric stall force, indirect	Schmitz *et al*. [14] (*M* in Holcomb-Wygle *et al*. [38])
10	KI	M1	Z	N	*Loligo pealeii* (squid)	Mo	kinesin (optic lobe)	10^−12^	N	34	5.50	162	R	stall force	Svoboda & Block [39]
11	KI	M1	Z	N	*Loligo pealeii* (squid)	Mo	kinesin	10^−12^	N	34	6.50	191	—	maximum stall force	Visscher *et al*. [40], Schnitzer *et al*. [15]
12	KI	M1	Z	N	*Bos taurus* (cow)	Ma	kinesin (brain)	10^−11^	N	34	6.70	197	26	uniform stall force	Higushi *et al*. [41]
13	KI	M1	Z	N	*Bos taurus* (cow)	Ma	kinesin (brain)	10^−11^	N	34	4.50	132	30	near isometric	Hunt *et al*. [42]
14	KI	M1	Z	N	*Bos taurus* (cow)	Ma	kinesin (brain)	10^−11^	N	34	5.40	159	25	force to stop single molecule	Meyhöfer & Howard [43]
15	KI	M1	Z	N	*Bos taurus* (cow)	Ma	kinesin (brain)	10^−11^	N	34	7	206	26	stall force	Kojima *et al*. [44]
16	KI	M1	Z	N	*Homo sapiens* (man)	Ma	kinesin-1 (recombinant)	10^−11^	N	34	7.60	224	—	single-kinesin maximum force	Jamison *et al*. [45]
17	MY	M1	Z	S	*Rana esculenta* (frog)	Am	myosin (tibialis anterior muscle)	5 × 10^−8^	N	36	3.60	100	4	isometric, indirect	Linari *et al*. [46]
18	MY	M1	Z	S	*Rana esculenta* (frog)	Am	Actomyosin (tibialis anterior m.)	5 × 10^−8^	N	36	10	278	4	indirect isometric (indep. n)	Piazzesi *et al*. [47]
19	MY	M1	Z	S	*Rana esculenta* (frog)	Am	myosin (tibialis anterior muscles)	5 × 10^−8^	N	36	5.70	158	4	indirect isometric (dep. on n)	Piazzesi *et al*. [48]
20	MY	M1	Z	T	*Oryctolagus cuniculus* (rabbit)	Ma	myosin (heavy meromyosin, ske. m.)	5 × 10^−8^	N	36	3.50	97	—	average isometric force	Finer *et al*. [49]
21	MY	M1	Z	T	*Oryctolagus cuniculus* (rabbit)	Ma	myosin (skeletal muscle)	5 × 10^−8^	N	36	5.70	158	27	peak isometric	Ishijima *et al*. [50]
22	MY	M1	Z	T	*Oryctolagus cuniculus* (rabbit)	Ma	myosin (heavy meromyosin, ske. m.)	5 × 10^−8^	N	36	3.30	92	R	direct (not isometric)	Miyata *et al*. [51]
23	MY	M1	Z	T	*Oryctolagus cuniculus* (rabbit)	Ma	myosin (psoas, fast skeletal m.)	5 × 10^−8^	N	36	6.30	175	32	indirect	Tsaturyan *et al*. [52]
24	MY	M1	Z	T	*Oryctolagus cuniculus* (rabbit)	Ma	myosin (skeletal white muscle)	5 × 10^−8^	N	36	6.50	181	R	direct (sliding not isometric)	Nishizaka *et al*. [53]
25	MY	M1	Z	T	*Oryctolagus cuniculus* (rabbit)	Ma	myosin (skeletal white muscle)	5 × 10^−8^	N	36	9.20	256	R	single molecule unbinding force	Nishizaka *et al*. [54]
26	MY	M1	Z	T	*Oryctolagus cuniculus* (rabbit)	Ma	Actomyosin (skeletal muscle)	5 × 10^−8^	N	36	9	250	—	direct isometric	Takagi *et al*. [55]
27	MY	M1	Z	T	*Oryctolagus cuniculus* (rabbit)	Ma	myosin (psoas)	5 × 10^−8^	N	36	6.30	175	32	indirect	
28	SP	M2	U	T	*Vorticella convallaria*	Pr	spasmoneme	6.8 × 10^−11^	N	1.2 × 10^6^	4 × 10^4^	33	—	maximum isometric tension	Moriyama *et al*. [56]
29	SP	M2	U	T	*Vorticella convallaria*	Pr	spasmoneme	6.8 × 10^−11^	N	2.0 × 10^6^	7 × 10^4^	35	—	not isometric tension	Upadhyaya *et al*. [12]
30	SP	M2	U	T	*Vorticella convallaria*	Pr	spasmoneme	6.8 × 10^−11^	N	2.0 × 10^6^	2.5 × 10^5^	125	—	isometric tension	Ryu *et al*. [57]
31	PI	M2	U	T	*Escherichia coli*	Ba	pili type P	10^−15^	N	46	27	587	—	optical tweezers, unfolding f.	Jass *et al*. [58]
32	PI	M2	U	T	*Escherichia coli*	Ba	pili type P	10^−15^	N	46	27	587	—	optical tweezers	Fällman *et al*. [59]
33	PI	M2	U	T	*Escherichia coli*	Ba	pili type P	10^−15^	N	46	28	609	—	isometric force	Andersson *et al*. [60]
34	PI	M2	U	T	*Escherichia coli*	Ba	pili type P	10^−15^	N	46	35	761	—	atomic f. microscopy, plateau	Miller *et al*. [11]
35	PI	M2	U	T	*Escherichia coli*	Ba	pili type I	10^−15^	N	48	60	1250	—	atomic force microscopy	Miller *et al*. [11]
36	PI	M2	U	T	*Neisseria gonorrhoeae*	Ba	pili type IV	10^−15^	Y	36	70	1944	—	detachment force	Biais *et al*. [10] (*M* in Kaiser [61], Merz *et al*. [62])
rotary motors															
37	FA	M2	U	N	*Escherichia coli*	Ba	F0 ATPase (ionic pump)	1.3 × 10^−15^	N	46	40/3.5	248	—		Noji *et al*. [63], Sambongi *et al*. [7]
38	FA	M2	U	N	*Bacillus*	Ba	F1 ATPase	3 × 10^−15^	N	74	40/4.5	120	23		Yasuda *et al*. [8]
39	FL	M2	U	S	*Escherichia coli*	Ba	flagellum (basal + hook)	1.6 × 10^−15^	Y	650	4500/20	346	—	stall (or slow rotation)	Berry and Berg [64] (*M* in Berg [9,65])
40	FL	M2	U	S	*Vibrio alginolyticus*	Ba	flagellum	1.3 × 10^−15^	N	650	2100/20	162	—	stall torque	Sowa *et al*. [66]
41	FL	M2	U	S	*Salmonella*	Ba	flagellum	4 × 10^−15^	N	650	2100/20	162	23	torque at zero speed	Nakamura *et al*. [67]
42	FL	M2	U	S	*Streptococcus*	Ba	flagellum	2 × 10^−16^	N	650	2500/20	192	22	torque at zero speed	Lowe *et al*. [68]
myofibrils															
43	MF	M2	Z	T	*Mus musculus* (mouse)	Ma	psoas (fast skeletal m.)	10^−11^	N	—	—	91	20	single myofibril not stretched	Powers *et al*. [69]
44	MF	M2	Z	T	*Oryctolagus cuniculus* (rabbit)	Ma	psoas (fast skeletal m.)	5 × 10^−8^	N	—	—	265	5	not skinned, single or few	Tesi *et al*. [5]
45	MF	M2	Z	T	*Oryctolagus cuniculus* (rabbit)	Ma	psoas (fast skeletal m.)	5 × 10^−8^	N	—	—	186	10	bundle (1–3 myofibrils)	Telley *et al*. [70]
46	MF	M2	Z	T	*Oryctolagus cuniculus* (rabbit)	Ma	psoas (fast skeletal m.)	5 × 10^−8^	N	—	—	250	23	single or 2–3 myofibrils	Shimamoto *et al*. [71]
47	MF	M2	Z	S	*Rana* sp. (frog)	Am	tibialis anterior & sartorius	5 × 10^−8^	N	—	—	376	15	single myofibril	Colomo *et al*. [72]
48	MF	M2	Z	N	*Rana* sp.(frog)	Am	heart atrial myocyte	1.8 × 10^−12^	N	—	—	149	15	single myocyte (1–5 myofibrils)	Colomo *et al*. [72] (*M* in Brandt *et al*. [73])
49	MF	M2	Z	N	*Rana esculenta* (frog)	Am	heart atrial	1.8 × 10^−12^	Y	—	—	120	20	single myocyte (1–5 myofibrils)	Brandt *et al*. [73]
50	MF	M2	Z	N	*Rana esculenta* (frog)	Am	heart ventricle	3.5 × 10^−12^	Y	—	—	124	20	single myocyte (1–5 myofibrils)	Brandt *et al*. [73]
51	MF	M2	Z	N	*Mus musculus* (mouse)	Ma	heart left ventricle	10^−11^	N	—	—	119	10	bundle (2–6 myofibrils)	Kruger *et al*. [74]
52	MF	M2	Z	N	*Mus musculus* (mouse)	Ma	heart left ventricle	10^−11^	N	—	—	138	10	bundle (2–6 myofibrils)	Stehle *et al.* [75]
53	MF	M2	Z	N	*Cavia porcellus* (guinea pig)	Ma	heart left ventricle	10^−11^	N	—	—	161	10	bundle (2–6 myofibrils)	Stehle *et al.* [75]
54	MF	M2	Z	N	*Cavia porcellus* (guinea pig)	Ma	heart left ventricle	10^−11^	N	—	—	149	10	bundle (2–6 myofibrils)	Stehle *et al.* [76]
55	MF	M2	Z	N	*Cavia porcellus* (guinea pig)	Ma	heart left ventricular trabeculae	10^−11^	N	—	—	141	10	bundle (1–3 myofibrils)	Telley *et al*. [70]
56	MF	M2	Z	N	*Cavia porcellus* (guinea pig)	Ma	heart left ventricle	10^−11^	N	—	—	196	10	bundle (2–6 myofibrils)	Stehle *et al*. [77]
57	MF	M2	Z	N	*Oryctolagus cuniculus* (rabbit)	Ma	heart right ventricle	10^−11^	N	—	—	145	21	single myofibril	Linke *et al*. [78]
58	MF	M2	Z	N	*Homo sapiens* (human)	Ma	heart left ventricle	10^−11^	N	—	—	151	10	bundle (2–6 myofibrils)	Stehle *et al.* [75]

For non-molecular motors the tensions (*f* = *F*/*A*) were always given in the articles cited.

All tensions were expressed in kilopascal. In papers giving several values or minimum and maximum, their mean was calculated. Values from different papers were never pooled. In tables 3 (molecular motors) and 4 (non-molecular motors) tensions given by different authors in different conditions for the same motor are listed separately (329 values). If the same motor of the same species, studied by different authors or the same authors in different conditions, are counted only once, the number of different motors is approximately 265 (the uncertainty arises from a few measurements in table 4 which were made on a mixture of distinct fibres or several muscles together).

**Table 4.** Non-molecular motors. (Same columns as in table 3. I, mass indicated in the cited article: Y = yes, N = no, R = indicated as a range (mean is given). Motor: f. fibre, m. muscle, DDF deep digital flexor, EDL extensor digitorum longue, Gastr. gastrocnemius, SDF superficial digital flexor, VI vastus intermedius, VL vastus lateralis, VM vastus medialis. Comment: f. fibre, m. muscle.)

no.	Ty	C	species	group	motor	*M* (kg)	*I*	*f* (kPa)	*T* (°C)	comment	reference
fibres											
1	FI	F	*Drosophila melanogaster* (fruit fly)	In	indirect flight muscle	1.9 × 10^−6^	N	3.6	15	skinned f., active isometric	Wang *et al*. [79]
2	FI	S	*Nephrops norvegicus* (lobster)	Cr	superficial flexor m. 1st abdominal segment (slow S1)	0.50	N	105	22	skinned single f.	Holmes *et al*. [80]
3	FI	S	*Nephrops norvegicus* (lobster)	Cr	superficial flexor m. 1st abdominal segment (slow S2)	0.50	N	31	22	skinned single f.	Holmes *et al*. [80]
4	FI	S	*Procambarus clarkii* (crayfish)	Cr	superficial abdominal extensor	0.05	N	430	20	not skinned single f.	Tameyasu [81]
5	FI	F	*Bombus lucorum* + *B. terrestris* (bumblebee drone + worker)	In	dorsal longitudinal flight m. (asynchronous)	5 × 10^−4^	N	55	40	skinned single f.	Gilmour & Ellington [82]
6	FI	S	*Carangus melampygus* (blue crevally, Pacific)	Fi	red f.	0.30	Y	43	25	skinned single f.	Johnston & Brill [83]
7	FI	S	*Carangus melampygus* (blue crevally, Pacific)	Fi	white f.	0.30	Y	183	25	skinned single f.	Johnston & Brill [83]
8	FI	S	*Chaenocephalus aceratus* (ice fish, Antartic)	Fi	myotomal m. fast f., −2 + 2°	1.03	Y	231	−1	skinned single f.	Johnston & Altringham [84]
9	FI	S	*Euthynuus affinis* (kawakawa, Pacific ocean)	Fi	red f.	3.20	Y	25	30	skinned single f.	Johnston & Brill [83]
10	FI	S	*Euthynuus affinis* (kawakawa, Pacific ocean)	Fi	white f.	3.20	Y	188	30	skinned single f.	Johnston & Brill [83]
11	FI	S	*Gadus morhua* (North Sea cod, temperate)	Fi	myotomal m. fast f., 2–12°	84	Y	187	8	skinned single f.	Johnston & Altringham [84]
12	FI	S	*Gadus morhua* (cod)	Fi	myotomal m. white f. (fast)	84	N	83	8	skinned single f.	Altringham & Johnston [85]
13	FI	S	*Gadus morhua* (cod)	Fi	myotomal m. red f. (slow)	84	N	186	8	skinned 2–6 f.	Altringham & Johnston [85]
14	FI	S	*Katsuwonus pelamis* (skipjack tuna, Pacific)	Fi	white f.	1.20	Y	157	25	skinned single f.	Johnston & Brill [83]
15	FI	S	*Katsuwonus pelamis* (skipjack tuna, Pacific)	Fi	red f.	1.20	Y	24	25	skinned single f.	Johnston & Brill [83]
16	FI	S	*Makaira nigricans* (Pacific blue marlin, tropical)	Fi	myotomal m. fast f., 10–30°	1.90	Y	156	20	skinned single f.	Johnston & Altringham [84]
17	FI	S	*Makaira nigricans* (Pacific Blue marlin)	Fi	white f.	85	R	176	25	skinned single f.	Johnston & Salamonski [86]
18	FI	S	*Makaira nigricans* (Pacific Blue marlin)	Fi	red f.	85	R	57	25	skinned 2–3 f.	Johnston & Salamonski [86]
19	FI	S	*Mugil cephalus* (grey mullet, Pacific reefs)	Fi	red f. (slow)	1.14	Y	52	20	skinned single f.	Johnston & Brill [83]
20	FI	S	*Mugil cephalus* (grey mullet, Pacific reefs)	Fi	white f.	1.14	Y	210	20	skinned single f.	Johnston & Brill [83]
21	FI	S	*Notothenia neglecta* (Antarctic fish)	Fi	white f. (fast)	0.60	Y	225	0	skinned single f.	Johnston & Brill [83]
22	FI	S	*Scorpaena notata* (Mediterranean fish)	Fi	anterior abdominal m. (fast f.)	0.023	Y	239	20	not skinned $	Wakeling & Johnston [87]
23	FI	S	*Scyliorhinus canicula* (dogfish)	Fi	myotomal m. red f. (slow)	35	N	82	8	skinned 2–6 f.	Altringham & Johnston [85]
24	FI	S	*Scyliorhinus canicula* (dogfish)	Fi	myotomal m. white f. (fast)	35	N	183	8	skinned single f.	Altringham & Johnston [85]
25	FI	S	*Xenopus laevis* (clawed frog)	Am	iliofibularis m. (slow f.)	0.10	N	300	22	not skinned single f.	Lännergren [88,89] (in Medler [4])
26	FI	S	*Pseudemys scripta elegans* (freshwater terrapin)	Re	iliofibularis pale thick f. (fast glycolytic)	0.30	Y	183	15	skinned single f.	Mutungi & Johnston [90]
27	FI	S	*Pseudemys scripta elegans* (freshwater terrapin)	Re	iliofibularis medium thick f.(fast oxidative glycolytic)	0.30	Y	120	15	skinned single f	Mutungi & Johnston [90]
28	FI	S	*Pseudemys scripta elegans* (freshwater terrapin)	Re	iliofibularis red thin (slow oxidative)	0.30	Y	71	15	skinned single f.	Mutungi & Johnston [90]
29	FI	F	*Calypte anna* (hummingbird)	Bi	pectoralis	4.7 × 10^−3^	Y	12	20	single fibre	Reiser *et al*. [91]
30	FI	F	*Calypte anna* (hummingbird)	Bi	ankle extensor	4.7 × 10^−3^	Y	94	20	single fibre	Reiser *et al*. [91]
31	FI	F	*Gallus domesticus* (chicken white leghorn)	Bi	pectoralis major white or pale f.	1.50	N	165	15	skinned single f.	Reiser *et al*. [92]
32	FI	N	*Gallus domesticus* (chicken white leghorn)	Bi	pectoralis major red strip (<1%, fast f., wing closer)	1.50	N	174	15	skinned single f.	Reiser *et al*. [92]
33	FI	F	*Gallus domesticus* (chicken white leghorn)	Bi	pectoralis major red strip (slow tonic f.)	1.50	N	126	15	skinned single f.	Reiser *et al*. [92]
34	FI	F	*Gallus domesticus* (chicken white leghorn)	Bi	anterior latissimus dorsi (slow tonic f.)	1.50	N	75	15	skinned single f.	Reiser *et al*. [92]
35	FI	F	*Taeniopygia guttata* (zebra finches)	Bi	pectoralis	4.7 × 10^−3^	Y	22	20	single fibre	Reiser *et al*. [91]
36	FI	F	*Taeniopygia guttata* (zebra finches)	Bi	ankle extensor	4.7 × 10^−3^	Y	79	20	single fibre	Reiser *et al*. [91]
37	FI	T	*Acinonyx jubatus* (cheetah)	Ma	gluteus, semitendinosus, longissimus m. (type 1)	41	Y	132	20	skinned fibre	West *et al*. [93]
38	FI	T	*Acinonyx jubatus* (cheetah)	Ma	gluteus, semitendinosus, longissimus m. (type 2)	41	Y	195	20	skinned fibre	West *et al*. [93]
39	FI	T	*Bos taurus* (cow Holstein)	Ma	usually soleus (slow f.)	160	Y	233	5.5	skinned single f.	Seow & Ford [94]
40	FI	T	*Bos taurus* (cow Angus-Hereford)	Ma	∼soleus (slow f.)	500	Y	60	5.5	skinned single f.	Seow & Ford [94]
41	FI	T	*Bos taurus* (cow Holstein)	Ma	usually extensor digitorum longue (fast f.)	160	Y	248	5.5	skinned single f.	Seow & Ford [94]
42	FI	T	*Bos taurus* (cow Angus-Hereford)	Ma	∼extensor digitorum longue (fast f.)	500	Y	88	5.5	skinned single f.	Seow & Ford [94]
43	FI	T	*Caracal caracal* (caracal)	Ma	vastus lateralis (type 2x)	15	N	211	12	single fibre	Kohn & Noakes [95]
44	FI	T	*Equus caballus* (horse)	Ma	soleus (type 1, 23% of m.)	420	Y	84	15	skinned single f.	Rome *et al*. [96]
45	FI	T	*Equus caballus* (horse)	Ma	soleus (type 2a, 43%)	420	Y	97	15	skinned single f.	Rome *et al*. [96]
46	FI	T	*Equus caballus* (horse)	Ma	soleus (type 2b, 34%)	420	Y	120	15	skinned single f.	Rome *et al*. [96]
47	FI	T	*Homo sapiens* (human cyclists)	Ma	vastus lateralis (type 1)	70	N	66	12	single fibre	Kohn & Noakes [95]
48	FI	T	*Homo sapiens* (human cyclists)	Ma	vastus lateralis (type 2a)	70	N	113	12	single fibre	Kohn & Noakes [95]
49	FI	T	*Homo sapiens* (human cyclists)	Ma	vastus lateralis (type 2ax)	70	N	155	12	single fibre	Kohn & Noakes [95]
50	FI	T	*Homo sapiens* (human male 25–45 yr)	Ma	vastus lateralis (slow type 1)	70	N	44	12	skinned single f.	Bottinelli *et al*. [97]
51	FI	T	*Homo sapiens* (human male 25–45 yr)	Ma	vastus lateralis (fast type 2)	70	N	61	12	skinned single f.	Bottinelli *et al*. [97]
52	FI	T	*Homo sapiens* (human male & female)	Ma	quadriceps vastus lateralis and soleus (type 1)	65	N	210	15	skinned single f.	Larsson & Moss [98]
53	FI	T	*Homo sapiens* (human male & female)	Ma	quadriceps vastus lateralis and soleus (type 2a fast)	65	N	200	15	skinned single f.	Larsson & Moss [98]
54	FI	T	*Homo sapiens* (human male & female)	Ma	quadriceps vastus lateralis and soleus (type 2b fast)	65	N	190	15	freeze-dried single f.	Larsson & Moss [98]
55	FI	T	*Macaca mulatta* (rhesus monkey)	Ma	soleus (slow type 1)	4	Y	180	15	skinned single f.	Fitts *et al*. [99]
56	FI	T	*Macaca mulatta* (rhesus monkey)	Ma	medial gastrocnemius (slow type 1)	4		180	15	skinned single f.	Fitts *et al*. [99]
57	FI	T	*Macaca mulatta* (rhesus monkey)	Ma	medial gastrocnemius (fast type 2)	4	Y	184	15	skinned single f.	Fitts *et al*. [99]
58	FI	T	*Mus musculus* (mouse CD1 male)	Ma	tibialis ant., gastrocnemius, soleus (fast f.)	0.04	R	70	12	skinned single f.	Pellegrino *et al*. [100]
59	FI	T	*Mus musculus* (mouse CD1 male)	Ma	tibialis ant., gastrocnemius, soleus (slow f.)	0.04	R	62	12	skinned single F.	Pellegrino *et al*. [100]
60	FI	T	*Mus musculus* (mouse CBA/J)	Ma	extensor digitorum longue (fast)	0.02	Y	153	5.5	skinned single f.	Seow & Ford [94]
61	FI	T	*Mus musculus* (mouse CBA/J)	Ma	soleus (slow)	0.02	Y	213	5.5	skinned single f.	Seow & Ford [94]
62	FI	T	*Oryctolagus cuniculus* (rabbit New Zealand male)	Ma	tibialis ant., gastr., soleus, EDL, VL, psoas (slow f.)	3.15	R	45	12	skinned single f.	Pellegrino *et al*. [100]
63	FI	T	*Oryctolagus cuniculus* (rabbit New Zealand male)	Ma	tibialis ant., gastr., soleus, EDL, VL, psoas (fast f.)	3.15	R	55	12	skinned single f.	Pellegrino *et al*. [100]
64	FI	T	*Oryctolagus cuniculus* (rabbit)	Ma	tibialis anterior (type 2a)	2.5	N	140	20	single f.	Sweeney *et al*. [101] in Schiaffino & Reggiani [102]
65	FI	T	*Oryctolagus cuniculus* (rabbit)	Ma	tibialis anterior (type 2b)	2.5	N	152	20	single f.	Sweeney *et al*. [101] in Schiaffino & Reggiani [102]
66	FI	T	*Oryctolagus cuniculus* (rabbit New Zealand white)	Ma	psoas (type 2b)	2.5	R	125	12	skinned single f.	Sweeney *et al*. [103]
67	FI	T	*Oryctolagus cuniculus* (rabbit New Zealand white)	Ma	tibialis anterior (type 2b)	2.5	R	120	12	skinned single f.	Sweeney *et al*. [103]
68	FI	T	*Oryctolagus cuniculus* (rabbit New Zealand white)	Ma	tibialis anterior (type 2a chronic stim)	2.5	R	100	12	skinned single f.	Sweeney *et al*. [103]
69	FI	T	*Oryctolagus cuniculus* (rabbit New Zealand white)	Ma	vastus intermedius (type 2a)	2.5	R	109	12	skinned single f.	Sweeney *et al*. [103]
70	FI	T	*Oryctolagus cuniculus* (rabbit New Zealand white)	Ma	soleus (type 1)	2.5	R	107	12	skinned single f.	Sweeney *et al*. [103]
71	FI	T	*Oryctolagus cuniculus* (rabbit New Zealand white male)	Ma	plantaris (slow)	2.5	N	251	15	skinned single f.	Greaser *et al*. [104]
72	FI	T	*Oryctolagus cuniculus* (rabbit New Zealand white male)	Ma	plantaris (intermediate)	2.5	N	253	15	skinned single f.	Greaser *et al.* [104]
73	FI	T	*Oryctolagus cuniculus* (rabbit New Zealand white male)	Ma	plantaris (fast)	2.5	N	249	15	skinned single f.	Greaser *et al*. [104]
74	FI	T	*Oryctolagus cuniculus* (rabbit New Zealand white)	Ma	extensor digitorum longue (fast)	2	Y	123	5.5	skinned single f.	Seow & Ford [94]
75	FI	T	*Oryctolagus cuniculus* (rabbit New Zealand white)	Ma	soleus (slow)	2	Y	147	5.5	skinned single f.	Seow & Ford [94]
76	FI	N	*Oryctolagus cuniculus* (rabbit)	Ma	diaphragam	5 × 10^−8^	N	99	20	single fibre	Reiser *et al*. [91]
77	FI	T	*Oryctolagus cuniculus* (rabbit)	Ma	psoas muscle (type 2x)	5 × 10^−8^	N	195	20	single fibre	Reiser *et al*. [91]
78	FI	T	*Ovis aries* (sheep)	Ma	∼extensor digitorum longue (fast)	55	Y	159	5.5	skinned single f.	Seow & Ford [94]
79	FI	T	*Ovis aries* (sheep)	Ma	∼soleus (slow)	55	Y	198	5.5	skinned single f.	Seow & Ford [94]
80	FI	T	*Panthera leo* (lion)	Ma	vastus lateralis (type 1)	180	N	162	12	single fibre	Kohn & Noakes [95]
81	FI	T	*Panthera leo* (lion)	Ma	vastus lateralis (type 2x)	180	N	191	12	single fibre	Kohn & Noakes [95]
82	FI	T	*Rattus norvegicus* (rat Wistar male)	Ma	tibialis anterior, plantaris, soleus (hindlimb, type 1)	0.25	N	68	12	skinned single f.	Bottinelli *et al*. [105]
83	FI	T	*Rattus norvegicus* (rat Wistar male)	Ma	tibialis anterior, plantaris, soleus (slow type 1)	0.35	R	68	12	skinned single f.	Pellegrino *et al*. [100]
84	FI	T	*Rattus norvegicus* (rat Wistar male)	Ma	tibialis anterior, plantaris, soleus (hindlimb, type 2a)	0.25	N	111	12	skinned single f.	Bottinelli *et al*. [105]
85	FI	T	*Rattus norvegicus* (rat Wistar male)	Ma	tibialis anterior, plantaris, soleus (hindlimb, type 2x)	0.25	N	95	12	skinned single f.	Bottinelli *et al*. [105]
86	FI	T	*Rattus norvegicus* (rat Wistar male)	Ma	tibialis anterior, plantaris, soleus (hindlimb, type 2b)	0.25	N	82	12	skinned single f.	Bottinelli *et al*. [105]
87	FI	T	*Rattus norvegicus* (rat Wistar male)	Ma	tibialis anterior, plantaris, soleus (fast type 2)	0.35	R	96	12	skinned single f.	Pellegrino *et al*. [100]
88	FI	T	*Rattus norvegicus* (rat Holtzman female)	Ma	soleus red (slow f.)	0.165	N	223	27	skinned 2–6 f.	Sexton & Gersten [106]
89	FI	T	*Rattus norvegicus* (rat Hotzman)	Ma	medial gastrocnemius (fast f.)	0.165	R	235	27	skinned 3–6 f.	Sexton [107]
90	FI	T	*Rattus norvegicus* (rat Hotzman)	Ma	tibialis anterior	0.165	R	140	27	skinnes 3–6 f.	Sexton [107]
91	FI	T	*Rattus norvegicus* (rat Sprague-Dawley)	Ma	extensor digitorum longue (fast)	0.20	Y	123	5.5	skinned single f.	Seow & Ford [94]
92	FI	T	*Rattus norvegicus* (rat Sprague-Dawley)	Ma	soleus (slow)	0.20	Y	100	5.5	skinned single f.	Seow & Ford [94]
93	FI	N	*Rattus norvegicus* (rat Sprague-Dawley)	Ma	diaphragm (type 1)	0.20	N	78	—	skinned single f.	Eddinger & Moss [108] in Schiaffino & Reggiani [102]
94	FI	N	*Rattus norvegicus* (rat Sprague-Dawley)	Ma	diaphragm (type 2a)	0.20	N	102	—	skinned single f.	Eddinger & Moss [108] in Schiaffino & Reggiani [102]
95	FI	N	*Rattus norvegicus* (rat Sprague-Dawley)	Ma	diaphragm (type 2b)	0.20	N	130	—	skinned single f.	Eddinger & Moss [108] in Schiaffino & Reggiani [102]
96	FI	T	*Rattus norvegicus* (rat Sprague-Dawley male)	Ma	tibialis anterior (fast)	0.25	Y	123	20	single fibre	Reiser *et al*. [91]
97	FI	T	*Rattus norvegicus* (rat Sprague-Dawley male)	Ma	soleus (slow)	0.25	Y	122	20	single fibre	Reiser *et al*. [91]
muscles *in vitro* or dissociated											
98	MU	S	*Alloteuthis subulata* (squid)	Mo	mantle m., ventral	0.50	N	262	11	piece of mantle	Milligan *et al*. [109]
99	MU	S	*Argopecten irradians* (bay scallop)	Mo	anterior side striated adductor	0.03	Y	242	10	bundle	Olson & Marsh [110]
100	MU	S	*Sepia officinalis* (cuttlefish)	Mo	mantle m., ventral	0.50	N	226	11	piece of mantle	Milligan *et al*. [109]
101	MU	N	*Carcinus maenas* (crab male)	Cr	flagellum abductor m. (continuous action)	0.035	R	56	15	whole m. nerve stim	Stokes & Josephson [111]
102	MU	N	*Carcinus maenas* (crab male)	Cr	scaphognathite levator (pump water across gills)	0.019	R	120	15	whole m. nerve stim	Stokes & Josephson [111]
103	MU	S	*Homarus americanus* (lobster)	Cr	abdominal extensor (fast)	0.75	R	82	12	bundle 6 f. K + caffeine	Jahromi & Atwood [112]
104	MU	S	*Homarus americanus* (lobster)	Cr	abdominal extensor (slow)	0.75	R	442	12	bundle 6 f. K + caffeine	Jahromi & Atwood [112]
105	MU	N	*Homarus americanus* (lobster)	Cr	claw closer m. (crusher)	0.05	N	200	14	whole m. K + caffeine	Elner & Campbell [113] (*M* in Medler [4])
106	MU	N	*Homarus americanus* (lobster)	Cr	claw closer m. (closer)	0.05	N	300	14	whole m. K + caffeine	Elner & Campbell [113] (*M* in Medler [4])
107	MU	F	*Bombus terrestris* (bumblebee male)	In	dorsoventral flight m. (asynchronous)	2.5 × 10^−4^	R	38	30	whole m.	Josephson & Ellington [114]
108	MU	F	*Cotinus mutabilis* (beetle)	In	flight metathoracic basalar (asynchron. wing depressor)	1.4 × 10^−3^	Y	19	40	whole m.	Josephson *et al*. [115]
109	MU	F	*Libellula pulchella* (dragonfly male & female)	In	flight m.	5.9 × 10^−4^	N	120	28	whole m.	Fitzhugh & Marden [116] (*M* in Marden [117])
110	MU	F	*Manduca sexta* (hawkmoth summer-flying)	In	large dorsal longitudinal flight m.	1.6 × 10^−3^	Y	70	30	whole m.	Marden [117]
111	MU	F	*Neoconocephalus robustus* (katydid male)	In	flight & stridulation, mesothoracic	1.0 × 10^−4^	N	48	35	whole m.	Josephson [118]
112	MU	F	*Neoconocephalus robustus* (katydid male)	In	flight, metathoracic	1.0 × 10^−4^	N	137	35	whole m.	Josephson [118]
113	MU	F	*Neoconocephalus triops* (katydid male)	In	flight & stridulation, mesothoracic	1.0 × 10^−4^	N	58	35	whole m.	Josephson [118]
114	MU	F	*Neoconocephalus triops* (katydid male)	In	flight, metathoracic	1.0 × 10^−4^	N	126	35	whole m.	Josephson [118]
115	MU	F	*Operophtera bruceata* (moth male winter-flying)	In	large dorsal longitudinal flight m.	1.17 × 10^−5^	Y	139	18	whole m.	Marden [117]
116	MU	F	*Schistocerca americana* (locust)	In	flight metathoracic 2nd tergocoxal (synchronous)	5.0 × 10^−4^	N	363	25	whole m.	Malamud & Josephson [119]
117	MU	N	*Cyprinus carpio* (carp)	Fi	hyohyoideus white & red f.	0.15	N	115	20	bundle	Granzier *et al*. [120]
118	MU	S	*Cyprinus carpio* (carp)	Fi	red f.	0.15	N	116	15	bundle ∼100 f. nerve stim	Rome & Sosnicki [121]
119	MU	S	*Myoxocephalis scorpius* (sculpin)	Fi	white f., anterior + posterior	0.20	R	195	12	bundle 6–100 f.	James *et al*. [122]
120	MU	S	*Myoxocephalis scorpius* (sculpin)	Fi	myotomal m. (fast f.)	0.27	R	198	5	bundle 6–20 f.	James *et al*. [122]
121	MU	S	*Myoxocephalis scorpius* (sculpin)	Fi	fast	0.28	R	190	5	fast start escape	James *et al*. [122]
122	MU	S	*Notothenia coriiceps* (Antarctic cod)	Fi	myotomal m. (fast f.)	0.154	Y	185	0	bundle 5–12 f.	Franklin & Johnston [123]
123	MU	S	*Scyliorhinus canicula* (dogfish)	Fi	white myotomal m.	0.45	R	241	12	bundle 1–10 f.	Curtin & Woledge [124]
124	MU	T	*Scyliorhinus canicula* (dogfish)	Fi	white myotomal m.	0.47	N	295	11	bundle 11–14 f.	Lou *et al*. [125]
125	MU	S	*Stenotomus chrysops* (scup)	Fi	red myotomal m.	0.14	Y	197	20	bundle	Coughlin *et al*. [126]
126	MU	S	*Stenotomus chrysops* (scup)	Fi	pink myotomal m.	0.14	N	151	20	bundle	Coughlin *et al*. [126]
127	MU	T	*Ambystoma tigrinum nebulosum* (salamander)	Am	extensor iliotibialis pars anterior leg	8.62 × 10^−3^	Y	339	20	whole m.	Else & Bennet [127]
128	MU	T	*Bufo americanus* (toad)	Am	white iliofibularis	0.04	Y	260	35		Johnston & Gleeson [128] in Medler [4]
129	MU	T	*Bufo marinus* (cane toad)	Am	white iliofibularis	0.18	Y	260	30		Johnston & Gleeson [128] in Medler [4]
130	MU	T	*Bufo woodhousei* (toad)	Am	white iliofibularis	0.11	Y	260	30		Johnston & Gleeson [128] in Medler [4]
131	MU	N	*Hyla chrysoscelis* (tree frog male diploid)	Am	tensor chodarum (laryngeal muscle, call production)	1.0 × 10^−2^	N	55	25	whole muscle	McLister *et al*. [129]
132	MU	T	*Hyla chrysoscelis* (tree frog male diploid)	Am	sartorius (leg)	1.0 × 10^−2^	N	252	25	whole muscle	McLister *et al*. [129]
133	MU	N	*Hyla cinera* (tree frog male)	Am	tensor chodarum	1.0 × 10^−2^	N	181	25	whole muscle	McLister *et al*. [129]
134	MU	T	*Hyla cinera* (tree frog male)	Am	sartorius	1.0 × 10^−2^	N	285	25	whole muscle	McLister *et al*. [129]
135	MU	N	*Hyla versicolor* (tree frog male tetraploid)	Am	tensor chodarum	1.0 × 10^−2^	N	94	25	whole muscle	McLister *et al*. [129]
136	MU	T	*Hyla versicolor* (tree frog male tetraploid)	Am	sartorius	1.0 × 10^−2^	N	241	25	whole muscle	McLister *et al*. [129]
137	MU	T	*Osteopilus septentrionalis* (Cuban tree frog)	Am	sartorius	0.013	Y	244	20	whole muscle	Peplowski & Marsh [130]
138	MU	T	*Rana catesbeiana* (north American bullfrog male)	Am	abductor indicus longus (forelimb)	0.376	Y	285	22	whole m. nerve stim	Peters & Aulner [131]
139	MU	T	*Rana catesbeiana* (frog male)	Am	flexor carpi radialis (forelimb)	3.76 × 10^−4^	Y	156	22	whole m. nerve stim	Peters & Aulner [131]
140	MU	T	*Rana catesbeiana* (frog male)	Am	extensor carpi radialis (forelimb)	3.76 × 10^−4^	Y	237	22	whole m. nerve stim	Peters & Aulner [131]
141	MU	T	*Rana catesbeiana* (frog male)	Am	extensor carpi ulnaris (forelimb)	3.76 × 10^−4^	Y	176	22	whole m. nerve stim	Peters & Aulner [131]
142	MU	T	*Rana catesbeiana* (frog female)	Am	abductor indicus longus (forelimb)	4.29 × 10^−4^	Y	359	22	whole m. nerve stim	Peters & Aulner [131]
143	MU	T	*Rana catesbeiana* (frog female)	Am	flexor carpi radialis (forelimb)	4.29 × 10^−4^	Y	118	22	whole m. nerve stim	Peters & Aulner [131]
144	MU	T	*Rana catesbeiana* (frog female)	Am	extensor carpi radialis (forelimb)	4.29 × 10^−4^	Y	285	22	whole m. nerve stim	Peters & Aulner [131]
145	MU	T	*Rana catesbeiana* (frog female)	Am	extensor carpi ulnaris (forelimb)	4.29 × 10^−4^	Y	197	22	whole m. nerve stim	Peters & Aulner [131]
146	MU	T	*Rana esculenta* (frog)	Am	sartorius	0.03	N	217	0	whole muscle	Stienen *et al*. [132]
147	MU	T	*Rana pipiens* (leopard frog)	Am	semimembranosus	0.03	N	255	25	bundle ∼100 f.	Lutz & Rome [133]
148	MU	T	*Xenopus laevis* (African clawed frog)	Am	gastrocnemius (main locomotory muscle in frogs)	9.8 × 10^−3^	Y	200	25	cold acclimated isolated m.	Seebacher *et al*. [134]
149	MU	T	*Dipsosaurus dorsalis* (lizard, desert iguana)	Re	iliofibularis (fast-twitch glycolytic region)	0.02	R	214	40	bundle	Marsh [135]
150	MU	T	*Sceloporus occidentalis* (lizard)	Re	iliofibularis (fast glycolytic f.)	0.0137	Y	188	35	bundle	Marsh & Bennet [136]
151	MU	F	*Coturnix chinensis* (blue-breasted quail)	Bi	pectoralis m. (flight)	0.046	Y	131	40	bundle	Askew & Marsh [137]
152	MU	T	*Cavia porcellus* (guinea pig)	Ma	soleus	0.13	R	147	20	whole muscle	Asmussen & Maréchal [138]
153	MU	T	*Dipodomys spectabilis* (kangaroo rat)	Ma	gastrocnemius, plantaris, soleus (ankle extensor group)	0.11	Y	200	—	whole m. nerve stim	Perry *et al*. [139]
154	MU	T	*Dipodomys spectabilis* (kangaroo rat)	Ma	gastrocnemius + plantaris (soleus = 2%)	0.11	Y	200	30	whole m. nerve stim	Biewener *et al*. [140] in Ettema [141]
155	MU	T	*Felis silvestris* (cat)	Ma	gastrocnemius (25% slow S f.)	4	N	60	—	single m. unit	Burke & Tsairis [142], figure 4
156	MU	T	*Felis silvestris* (cat)	Ma	gastrocnemius (20% fast fatigue resistant FR f.)	4	N	270	—	single m. unit	Burke & Tsairis [142], figure 4
157	MU	T	*Felis silvestris* (cat)	Ma	gastrocnemius (55% fast fatigable FF f.)	4	N	172	—	single m. unit	Burke & Tsairis [142], figure 4
158	MU	F	*Murina leucogaster* (korean bat)	Ma	biceps brachii	7.6 × 10^−3^		155	25		Choi *et al*. [143] in Medler [4]
159	MU	T	*Mus musculus* (mouse NMRI)	Ma	soleus	0.035	R	148	20	whole muscle	Asmussen & Maréchal [138]
160	MU	T	*Mus musculus* (mouse 129/Re male)	Ma	soleus	0.02	N	154	37	whole muscle	Rowe [144]
161	MU	T	*Mus musculus* (mouse 129/Re female)	Ma	soleus	0.02	N	211	37	whole muscle	Rowe [144]
162	MU	N	*Mus musculus* (mouse albino female)	Ma	diaphragm	0.03	R	176	35	1 mm strip	Luff [145]
163	MU	N	*Mus musculus* (mouse albino female)	Ma	inferior rectus	0.03	R	102	35	whole muscle	Luff [145]
164	MU	T	*Mus musculus* (mouse albino female)	Ma	extensor digitorum longus	0.03	R	249	35	whole muscle	Luff [145]
165	MU	T	*Mus musculus* (mouse albino female)	Ma	soleus	0.03	R	211	35	whole muscle	Luff [145]
166	MU	T	*Mus musculus* (mouse Swiss female)	Ma	soleus (slow twitch m.)	0.02	N	212	21	bundle	Barclay *et al*. [146]
167	MU	T	*Mus musculus* (mouse Swiss female)	Ma	extensor digitorum longue EDL (fast)	0.02	N	180	21	bundle	Barclay *et al*. [146]
168	MU	T	*Mus musculus* (mouse female)	Ma	extensor digitorum longus (2a + 2b f.)	0.026	Y	243	37	whole muscle	Askew & Marsh [147]
169	MU	T	*Mus musculus* (mouse female)	Ma	soleus (2a fast oxida glycolyt + 1 slow oxida)	0.026	Y	269	37	whole muscle	Askew & Marsh [147]
170	MU	T	*Notomys alexis* (hopping mouse)	Ma	gastrocnemius	0.03	Y	238	30	whole muscle	Ettema [141]
171	MU	N	*Oryctolagus cuniculus* (rabbit)	Ma	extraocular inferior oblique	2.80	Y	39	35	whole muscle	Asmussen *et al*. [148]
172	MU	T	*Rattus norvegicus* (rat male Fisher 344)	Ma	medial gastrocnemius (slow S f.)	0.46	R	167	36	motor unit nerve stim	Kanda & Hashizume [149]
173	MU	T	*Rattus norvegicus* (rat male Fisher 344)	Ma	medial gastrocnemius (fast fatigue resistant FR f.)	0.46	R	214	36	motor unit nerve stim	Kanda & Hashizume [149]
174	MU	T	*Rattus norvegicus* (rat male Fisher 344)	Ma	medial gastrocnemius (fast fatigable FF f.)	0.46	R	251	36	motor unit nerve stim	Kanda & Hashizume [149]
175	MU	T	*Rattus norvegicus* (rat)	Ma	medial gastrocnemius	0.31	Y	209	30	whole muscle	Ettema [141]
176	MU	T	*Rattus norvegicus* (rat Wistar female)	Ma	extensor digitorum longue (tetanic, normal)	0.28	Y	281	—	whole m. nerve stim	Close [150]
177	MU	T	*Rattus norvegicus* (rat Wistar female)	Ma	extensor digitorum longue (tetanic, normal)	0.25	Y	294	35	whole m. nerve stim	Bárány & Close [151]
178	MU	T	*Rattus norvegicus* (rat male)	Ma	extensor digitorum longue (fast twitch)	0.20	N	360	35	bundle	Ranatunga [152]
179	MU	T	*Rattus norvegicus* (rat Wistar female)	Ma	soleus (tetanic, normal)	0.275	Y	189	—	whole m. nerve stim	Close [150]
180	MU	T	*Rattus norvegicus* (rat Wistar female)	Ma	soleus (tetanic, normal, mean oper. I-II-III)	0.25	Y	206	35	whole m. nerve stim	Bárány & Close [151]
181	MU	T	*Rattus norvegicus* (rat)	Ma	soleus (slow)	0.20	N	223	35	strip	Ranatunga [152]
182	MU	T	*Rattus norvegicus* (white rat)	Ma	gastrocnemius, plantaris, soleus (ankle extensor group)	0.24	Y	206	37	whole m. nerve stim	Perry *et al*. [139]
183	MU	N	*Rattus norvegicus* (rat)	Ma	diaphragm	0.20	N	159	37	strip 5–11 mm + nerve st	Goffart & Ritchie [153]
184	MU	N	*Rattus norvegicus* (rat)	Ma	diaphragm	0.30		205	26		Johnson *et al*. [154] in Medler [4]
185	MU	T	*Rattus norvegicus* (rat Wistar)	Ma	soleus	0.25	R	168	20	whole muscle	Asmussen & Maréchal [138]
186	MU	T	*Thylogale billiardieri* (wallaby red-bellied pademelon)	Ma	gastrocnemius medial head	5.00	R	200	32	whole m. nerve stim	Morgan *et al*. [155] in Ettema [141]
muscles *in vivo*											
187	MV	N	*Callinectes sapidus* (blue crab)	Cr	claw closer (crusher)	0.165	R	638	10	crushing	Govind & Blundon [156]
188	MV	N	*Callinectes sapidus* (blue crab)	Cr	claw closer (cutter)	0.165	R	514	10	cutting	Govind & Blundon [156]
189	MV	N	*Cancer antennarius* (crab)	Cr	claw closer N	0.112	Y	866	11	biting	Taylor [157]
190	MV	N	*Cancer branneri* (crab)	Cr	claw closer N	0.030	Y	1031	11	biting	Taylor [157]
191	MV	N	*Cancer gracilis* (crab)	Cr	claw closer N	0.156	Y	525	11	biting	Taylor [157]
192	MV	N	*Cancer magister* (crab)	Cr	claw closer N	0.310	Y	756	11	biting	Taylor [157]
193	MV	N	*Cancer oregonensis* (crab)	Cr	claw closer N	0.014	Y	1007	11	biting	Taylor [157]
194	MV	N	*Cancer productus* (crab)	Cr	claw closer N	0.136	Y	792	11	biting	Taylor [157]
195	MV	N	*Menippe mercenaria* (stone crab)	Cr	claw closer (crusher chela)	0.25	N	740	30	squeezing	Blundon [158] (*M* in Medler [4])
196	MV	N	*Menippe mercenaria* (stone crab)	Cr	claw closer (cutter chela)	0.25	N	785	30	squeezing	Blundon [158] (*M* in Medler [4])
197	MV	N	*Archegozetes longisetosus* (mite)	Ar	claws	1.0 × 10^−7^	Y	1200	—	holding	Heethoff & Koerner [159]
198	MV	T	*Athous haemorrhoidalis* (click beetle)	In	M4 jumping m.	40 × 10^−6^	Y	700	>25	jumping	Evans [160]
199	MV	T	*Carabus problematicus* (click beetle)	In	femoral rotator m. (hind leg)	0.35 × 10^−3^	Y	210	23	pushing	Evans [161]
200	MV	N	*Cyclommatus metallifer* (stag beetle male)	In	mandible closer muscles	1.36 × 10^−3^	Y	180	22	biting	Goyens *et al*. [162]
201	MV	F	*Drosophila hydei* (fruit fly female)	In	flight m.	1.90 × 10^−6^	N	40	—	tethered flight	Dickinson & Lighton [163]
202	MV	T	*Schistocerca gregaria* (locust female)	In	extensor tibiae (metathoracic leg)	3 × 10^−3^	R	700	30	jumping	Bennet-Clark [164]
203	MV	T	*Spilopsyllus cuniculus* (rabbit flea)	In	metathoracic leg	0.45 × 10^−6^	Y	300	—	jumping	Bennet-Clark & Lucey [165]
204	MV	S	*Xenopus* (frog)	Am	plantaris longus	0.10		200	—	swimming	Richards unpublished in Biewener [166]
205	MV	T	*Anas platyrhynchos* (mallard duck)	Bi	lateral gastrocnemius m.	1.05	Y	126	40	walking	Biewener & Corning [167]
206	MV	S	*Anas platyrhynchos* (mallard duck)	Bi	lateral gastrocnemius m.	1.05	Y	62	40	swimming	Biewener & Corning [167]
207	MV	F	*Anas platyrhynchos* (mallard duck)	Bi	pectoralis	1.0	Y	236	40	ascending flight	Williamson *et al*. [168]
208	MV	F	*Columbia liva* (pigeon)	Bi	pectoralis (flight m.)	0.31	R	76	40	ascending flight	Dial & Biewener [169]
209	MV	T	*Numida meleagris* (guinea fowl)	Bi	digital flexor-IV (hind limb)	1.25	Y	115	—	jumping	Biewener [166]
210	MV	T	*Numida meleagris* (guinea fowl)	Bi	digital flexor-IV (hind limb)	1.25	Y	130	—	running	Daley & Biewener [170]
211	MV	T	*Numida meleagris* (guinea fowl)	Bi	lateral gastrocnemius (hind limb)	1.25	Y	133	—	Jumping	Biewener [166]
212	MV	T	*Numida meleagris* (guinea fowl)	Bi	lateral gastrocnemius (hind limb)	1.25	Y	39	—	running	Daley & Biewener [170]
213	MV	F	S*turnus vulgaris* (starling)	Bi	pectoralis, oxidative f.	0.072	Y	122	40	level flight	Biewener *et al*. [171]
214	MV	T	*Canis familiaris* (dog)	Ma	gastrocnemius + plantaris (ankle extensors)	36		310	—	jumping	Alexander [172]
215	MV	T	*Canis familiaris* (dog)	Ma	biceps femoris + 4 others (hip extensors)	36		270	—	jumping	Alexander [172]
216	MV	T	*Canis familiaris* (dog)	Ma	rectus femoris + VM + VL (knee extensors)	36		240	—	jumping	Alexander [172]
217	MV	T	*Canis familiaris* (dog)	Ma	triceps surae (elbow extensor)	36		290	—	jumping	Alexander [172]
218	MV	T	*Canis familiaris* (dog)	Ma	gastrocnemius, plantaris	36	Y	340	37	galloping 15.5 m s^−1^	Jayes & Alexander [173]
219	MV	T	*Canis familiaris* (dog)	Ma	biceps femoris + 4 others	36	Y	150	37	galloping 15.5 m s^−1^	Jayes & Alexander [173]
220	MV	T	*Canis familiaris* (dog)	Ma	sartorius, rectus femoris, tensor fasciae latae	36	Y	310	37	galloping 15.5 m s^−1^	Jayes & Alexander [173]
221	MV	T	*Canis familiaris* (dog)	Ma	rhomboideus	36	Y	300	37	galloping 15.5 m s^−1^	Jayes & Alexander [173]
222	MV	T	*Canis familiaris* (dog)	Ma	latissimus dorsi	36	Y	380	37	galloping 15.5 m s^−1^	Jayes & Alexander [173]
223	MV	T	*Canis familiaris* (dog)	Ma	pectoralis profundus	36	Y	260	37	galloping 15.5 m s^−1^	Jayes & Alexander [173]
224	MV	T	*Canis familiaris* (dog)	Ma	serratus ventralis thoracis	36	Y	300	37	galloping 15.5 m s^−1^	Jayes & Alexander [173]
225	MV	T	*Canis familiaris* (dog)	Ma	pectorales superficiales	36	Y	370	37	galloping 15.5 m s^−1^	Jayes & Alexander [173]
226	MV	T	*Capra hircus* (goat)	Ma	superficial digital flexor	34	Y	58	—	cantering	McGuigan *et al.* unpublished in Biewener [166]
227	MV	T	*Capra hircus* (goat)	Ma	gastrocnemius	34	Y	72	—	cantering	McGuigan *et al.* unpublished in Biewener [166]
228	MV	T	*Dipodomys spectabilis* (kangaroo rat)	Ma	gastrocnemius, plantaris, soleus (ankle extensor group)	0.11	Y	69	—	hopping 1.5 m s^−1^	Perry *et al*. [139]
229	MV	T	*Dipodomys spectabilis* (kangaroo rat)	Ma	ankle extensors	0.11	R	38	—	hopping slow 0.7 m s^−1^	Biewener *et al*. [140]
230	MV	T	*Dipodomys spectabilis* (kangaroo rat)	Ma	ankle extensors	0.11	R	105	—	hopping fast 1.9 m s^−1^	Biewener *et al*. [140]
231	MV	T	*Dipodomys spectabilis* (kangaroo rat)	Ma	triceps surae	0.11	Y	297	—	jumping peak force	Biewener & Blickhan [174] in Biewener [166]
232	MV	T	*Equus caballus* (horse)	Ma	fore DDF & fore SDF, gastrocnemius	275	Y	66	—	walking peak f	Biewener [175]
233	MV	T	*Equus caballus* (horse)	Ma	fore DDF & fore SDF, gastrocnemius	275	Y	107	—	trotting peak f	Biewener [175]
234	MV	T	*Equus caballus* (horse)	Ma	DDF, SDF, gastrocnemius	275	Y	157	—	galloping peak f	Biewener [175]
235	MV	T	*Equus caballus* (horse)	Ma	DDF, SDF, gastrocnemius	275	Y	240	—	highest stress	Biewener [175]
236	MV	T	*Felis silvestris* (cat)	Ma	plantaris, SDF	3.6	<	123	—	trotting	Biewener [166] based on Herzog *et al*. [176]
237	MV	T	*Felis silvestris* (cat)	Ma	gastrocnemius	3.6	<	73	—	trotting	Biewener [166] based on Herzog *et al*. [176]
238	MV	T	*Homo sapiens* (human)	Ma	triceps surae	76	Y	151	37	running 4 m s^−1^	Thorpe *et al*. [177]
239	MV	T	*Homo sapiens* (human)	Ma	quadriceps	76	Y	255	37	running 4 m s^−1^	Thorpe *et al*. [177]
240	MV	T	*Homo sapiens* (human)	Ma	hip extensors	76	Y	110	37	running 4 m s^−1^	Thorpe *et al*. [177]
241	MV	T	*Homo sapiens* (human)	Ma	triceps surae	76	Y	101	37	high jump	Thorpe *et al*. [177]
242	MV	T	*Homo sapiens* (human)	Ma	quadriceps	76	Y	277	37	high jump	Thorpe *et al*. [177]
243	MV	T	*Homo sapiens* (human)	Ma	hip extensors	76	Y	120	37	high jump	Thorpe *et al*. [177]
244	MV	T	*Homo sapiens* (human male & female)	Ma	quadriceps	69.5	Y	76	37	test chair before training	Rutherford & Jones [178]
245	MV	T	*Homo sapiens* (human male & female)	Ma	quadriceps	69.5	Y	82	37	test chair after training	Rutherford & Jones [178]
246	MV	T	*Homo sapiens* (human elderly 67.1 ± 2 yr)	Ma	vastus lateralis (knee)	73.5	Y	236	37	control pre-training	Reeves *et al*. [179]
247	MV	T	*Homo sapiens* (human elderly 67.1 ± 2 yr)	Ma	vastus lateralis (knee)	73.5	Y	215	37	control post-training	Reeves *et al*. [179]
248	MV	T	*Homo sapiens* (human elderly 74.3 ± 3.5 yr)	Ma	vastus lateralis (knee)	69.7	Y	270	37	test pre-training	Reeves *et al*. [179]
249	MV	T	*Homo sapiens* (human elderly 74.3 ± 3.5 yr)	Ma	vastus lateralis (knee)	69.7	Y	321	37	test post-training	Reeves *et al*. [179]
250	MV	T	*Homo sapiens* (human men 28.2 ± 3.6 yr)	Ma	quadriceps	78.8	Y	550	37	isokinetic dynamometer	O'Brien *et al*. [180]
251	MV	T	*Homo sapiens* (human women 27.4 ± 4.2 yr)	Ma	quadriceps	64	Y	573	37	isokinetic dynamometer	O'Brien *et al*. [180]
252	MV	T	*Homo sapiens* (human boys 8.9 ± 0.7 yr)	Ma	quadriceps	35.6	Y	540	37	isokinetic dynamometer	O'Brien *et al*. [180]
253	MV	T	*Homo sapiens* (human girls 9.3 ± 0.8 yr)	Ma	quadriceps	41.9	Y	598	37	isokinetic dynamometer	O'Brien *et al*. [180]
254	MV	T	*Homo sapiens* (human men)	Ma	biceps femoris + 4 others (knee)	61.3	Y	53	37	isokinetic dynamometer	Kanehisa *et al*. [181]
255	MV	T	*Homo sapiens* (human men)	Ma	quadriceps femoris (knee extensors)	61.3	Y	79	37	isokinetic dynamometer	Kanehisa *et al*. [181]
256	MV	T	*Homo sapiens* (human women)	Ma	knee flexors	58.5	Y	39	37	isokinetic dynamometer	Kanehisa *et al*. [181]
257	MV	T	*Homo sapiens* (human women)	Ma	knee extensors	58.5	Y	63	37	isokinetic dynamometer	Kanehisa *et al*. [181]
258	MV	T	*Homo sapiens* (human men)	Ma	biceps brachii & brachialis (elbow flexors)	61.3	Y	132	37	isokinetic dynamometer	Kanehisa *et al*. [181]
259	MV	T	*Homo sapiens* (human men)	Ma	triceps brachii (elbow extensors)	61.3	Y	111	37	isokinetic dynamometer	Kanehisa *et al*. [181]
260	MV	T	*Homo sapiens* (human women)	Ma	elbow flexors	58.5	Y	137	37	isokinetic dynamometer	Kanehisa *et al*. [181]
261	MV	T	*Homo sapiens* (human women)	Ma	elbow extensors	58.5	Y	110	37	isokinetic dynamometer	Kanehisa *et al*. [181]
262	MV	T	*Homo sapiens* (human men 28 ± 4 yr)	Ma	soleus	75	Y	150	37	isokinetic dynamometer	Maganaris *et al*. [182]
263	MV	T	*Homo sapiens* (human men 28 ± 4 yr)	Ma	tibialis anterior	75	Y	155	37	isokinetic dynamometer	Maganaris *et al*. [182]
264	MV	T	*Homo sapiens* (human males 34 ± 4.7 yr)	Ma	quadriceps vastus lateralis	74.1	Y	237	37	isometric voluntary contract.	Narici *et al*. [183]
265	MV	T	*Homo sapiens* (human males 34 ± 4.7 yr)	Ma	quadriceps vastus intermedius	74.1	Y	241	37	isometric volunt. contraction	Narici *et al*. [183]
266	MV	T	*Homo sapiens* (human males 34 ± 4.7 yr)	Ma	quadriceps vastus medialis	74.1	Y	279	37	isometric volunt. contraction	Narici *et al*. [183]
267	MV	T	*Homo sapiens* (human males 34 ± 4.7 yr)	Ma	quadriceps rectus femoris	74.1	Y	243	37	isometric volunt. contraction	Narici *et al*. [183]
268	MV	T	*Homo sapiens* (human males 38 ± 8 yr)	Ma	gastrocnemius medialis	67.8	Y	97	37	whole muscle + MRI	Narici *et al*. [183]
269	MV	T	*Homo sapiens* (human males 21.3 ± 3.4 yr)	Ma	quadriceps femoris	76.2	Y	297	37	max. volunt. contrac. (2 meth)	Erskine *et al*. [184]
270	MV	T	*Homo sapiens* (human young 22 yr)	Ma	triceps surae (ankle plantar flexor)	70		329	37	electrically evoked contract.	Davies *et al*. [185]
271	MV	T	*Homo sapiens* (human)	Ma	ankle plantar flexor	70	N	108	37	voluntary isometric torque	Fukunaga *et al*. [186]
272	MV	T	*Homo sapiens* (human)	Ma	ankle plantar flexor	70	N	382	37	external force	Haxton [187] in Maganaris *et al*. [182]
273	MV	T	*Homo sapiens* (human)	Ma	ankle plantar flexor	70	N	628	37	external force	Herman [188] in Maganaris *et al*. [182]
274	MV	T	*Homo sapiens* (human)	Ma	ankle plantar flexor	70	N	549	37	external force	Reys [189] in Maganaris *et al*. [182]
275	MV	T	*Homo sapiens* (human)	Ma	ankle plantar flexor	70	N	412	37	external force	Weber [190] in Maganaris *et al*. [182]
276	MV	T	*Loxodonta africana* (elephant)	Ma	knee quadriceps	2500	Y	140	37	running 4–4.5 m s^−1^	Alexander *et al*. [191]
277	MV	T	*Loxodonta africana* (elephant)	Ma	ankle extensors	2500	Y	140	37	running 4–4.5 m s^−1^	Alexander *et al*. [191]
278	MV	T	*Loxodonta africana* (elephant)	Ma	elbow triceps	2500	Y	140	37	running 4–4.5 m s^−1^	Alexander *et al*. [191]
279	MV	T	*Macropus eugenii* (tammar wallaby)	Ma	plantaris	4.8	Y	262	—	hopping 5.5 m s^−1^	Biewener & Baudinette [192]
280	MV	T	*Macropus eugenii* (tammar wallaby)	Ma	gastrocnemius	4.8	Y	227	—	hopping 5. m s^−1^	Biewener & Baudinette [192]
281	MV	T	*Macropus rufogriseus* (rock wallaby)	Ma	triceps surae	6.6	Y	279	—	jumping	McGowan & Biewener unpublished in Biewener [166]
282	MV	T	*Macropus rufogriseus* (rock wallaby)	Ma	triceps surae	6.6	Y	201	—	hopping	McGowan & Biewener unpublished in Biewener [166]
283	MV	T	*Macropus rufus* (red kangaroo juvenile)	Ma	plantaris + gastrocnemius (ankle extensors)	24	R	300	—	hopping	Alexander & Vernon [193]
284	MV	T	*Macropus rufus* (red kangaroo juvenile)	Ma	hip extensors	24	R	190	—	hopping	Alexander & Vernon [193]
285	MV	T	*Macropus rufus* (red kangaroo juvenile)	Ma	rectus femoris + VL + VI + VM (knee extensors)	24	R	240	—	hopping	Alexander & Vernon [193]
286	MV	T	*Protemnodon rufogrisea* (Bennett s wallaby)	Ma	plantaris + gastrocnemius (ankle extensors)	10.5	Y	150	—	hopping	Alexander & Vernon [193]
287	MV	T	*Protemnodon rufogrisea* (Bennett s wallaby)	Ma	hip extensors	10.5	Y	140	—	hopping	Alexander & Vernon [193]
288	MV	T	*Protemnodon rufogrisea* (Bennett s wallaby)	Ma	rectus femoris + VL + VI + VM (knee extensors)	10.5	Y	75	—	hopping	Alexander & Vernon [193]
289	MV	T	*Rattus norvegicus* (white rat)	Ma	gastrocnemius, plantaris, soleus (ankle extensors)	0.24	Y	70		galloping 1.5 m s^−1^	Perry *et al*. [139]
290	MV	T	*Syncerus caffer* (buffalo)	Ma	ankle extensors	500	Y	150	37	galloping 5 m s^−1^	Alexander *et al*. [191]
291	MV	T	*Syncerus caffer* (buffalo)	Ma	elbow triceps	500	Y	300	37	galloping 5 m s^−1^	Alexander *et al*. [191]

### 2.4. Other motor classifications

The data were also analysed with respect to the structure of motors, their function and the taxonomic position of the organisms.

For comparing structures, the original 13 types, from molecules to muscles, were aggregated in five classes (M1, M2, FI, MU, MV) or two classes (molecular M1 + M2 and non-molecular) as defined above. In some figures and table 5, MF, for which the cross-section was indicated in the articles cited, was shown separately from the other M2 motors.

**Table 5.** Summary statistics^a^ of specific tension *f* (in kPa) Per main motor types and functions.

		*n*	min	max	Q10	Q90	med.	IQR	mean	s.d.
motor types	all	349	4	1944	62	354	174	136	212	196
	all molecular	58	16	1944	72	524	160	129	239	303
	all non-molecular	291	4	1200	62	339	180	137	206	167
	PI	6	587	1944	587	1875	685	663	956	547
	non-PI	343	4	1200	62	312	167	134	199	158
motor types (except PI)	molecular	52	16	376	60	254	155	86	156	77
	non-molecular	291	4	1200	62	339	180	137	206	167
	M1	27	16	278	28	252	158	102	146	75
	M2^b^	9	33	346	34	307	162	107	158	99
	MF	16	91	376	119	264	149	60	173	71
	M2 + MF	25	33	376	91	265	149	70	167	80
	FI	97	4	430	53	230	123	105	136	73
	MU	89	19	442	75	285	200	98	195	81
	MV	105	38	1200	70	638	227	199	281	240
motor functions (except PI)	non-locomotor	55	16	1200	78	785	159	123	275	287
	locomotor	288	4	700	61	300	174	136	184	113
	swimming	53	18	442	50	282	183	131	169	98
	flying	25	4	363	19	165	79	87	100	78
	terrestrial	210	33	700	70	300	187	133	198	116

^a^Number of *f* values, minimum, maximum, quantile 10%, quantile 90%, median, interquartile range 25–75%, mean and standard deviation of *f*.

^b^This line M2 does not include myofibrils MF.

The functional groups were defined by the contribution of the motor to the overall movement of their parent organism, the four basic categories being swimming (Swim), flying (Fly), moving with respect to a solid surface (terrestrial Terr) and no direct contribution to locomotion (non-loc). Examples of non-loc motors are RNA polymerase, cytoplasmic dynein, kinesin, *F*_0_/*F*_1_-ATPase and various muscular motors (heart, diaphragm, wing closer, gill pump, claw closer, larynx, eye).

For taxonomic comparisons, groups 5 with number of *f* values less than 5 (protozoa, algae, fungi, echinoderms, arachnids) were excluded.

### 2.5. Body mass

Finally, the tensions were analysed with respect to the mass *M* of the ‘body’ that the motor contributes to move. For molecular motors this is the mass of the cell from which the motor was extracted. When not reported, cell masses were estimated from other sources or calculated from the cell size. In non-molecular motors, tensions were analysed with respect to the mass *M* of the corresponding animal. When not reported, body masses were also estimated from other sources. Note that as a consequence of these choices a different mass was used for a myosin molecule (molecular motor) and a muscle fibre (non-molecular motor) from the same organism. The organisms considered range in mass from the bacterium *Escherichia coli* (1.3 × 10^−15^ kg) to the muscular fibre (5 × 10^−8^ kg) for the cells, and from the mite *Archegozetes longisetosus* (10^−7^ kg) to the elephant (2500 kg) for the multicellular organisms.

For both *f* and *M*, means of a series of equivalent measurements by the same author(s) were preferred when available. When only minimum and maximum values were given, we took their mean.

### 2.6. Statistics

Statistical distributions were compared with the Kolmogorov–Smirnov test [194]. Multiple distributions were compared with the one-way analysis of variance (ANOVA) and corresponding multiple comparison of means using Tukey–Kramer adjustment. Slopes of least-square regressions of log_10_(*f*) versus log_10_(*M*) were compared with 0 using the *F* test. Details of statistical analyses are given as the electronic supplementary material, tables S1–S6 for ANOVA and multiple comparison of means and tables S7–S12 for regressions. All tests were performed with the Matlab Statistical Toolbox (The Mathworks, Natick, USA).

## 3. Results

The data have been analysed in terms of the maximum force per cross-sectional area *f*. We consider separately motors made of single molecules (denoted M1) and molecular assemblies (M2, MF) that we collectively call ‘molecular motors’, whereas the other motors, muscle fibres (FI) and whole muscles (MU for dissected muscles or MV for behaving animals) are called ‘non-molecular motors’. We have also analysed the data in terms of the mass *M* of the ‘body’ that the motor contributes to move and to whether the motor contributes to the overall movement of the parent organism.

The characteristic sizes of molecular motors are given in the table 2. All data (species, taxonomic group, motor type, motor function, motor description, cell or body mass *M*, comment on *M*, specific tension *f*, temperature, reference) are gathered in table 3 for molecular motors and table 4 for non-molecular motors. In table 3, *f* was calculated from the measured force or torque given in the references cited and the cross-sectional area and lever arm given in table 2. The statistics on *f* are summarized in table 5.

### 3.1. Specific tensions of molecular and non-molecular motors follow similar statistical distributions

**Figure 1.**
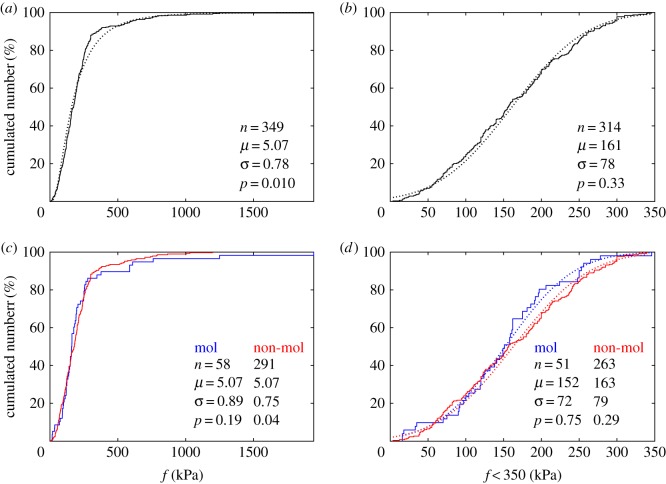
Distributions of specific tensions *f*. (*a*) Empirical cumulated distribution function (CDF). All *f* values are shown along the *x*-axis as stepwise increments, giving a complete and undistorted view of the original data. Empirical CDF is fitted to a lognormal distribution of mean *µ* and s.d. *σ* (dotted black line); fit is rejected at level 5% (*p* = 0.01). (*b*) Empirical CDF of *f* < 350 kPa (solid black line) with fitted normal distribution of *µ* and *σ* in kPa (dotted black line), not rejected at level 5% (*p* = 0.33). (*c*) Empirical CDFs of *f* for molecular motors (blue line, fitted lognormal not rejected) and non-molecular motors (red line, fitted lognormal rejected); the two distributions are not significantly different (*p* = 0.40). (*d*) Empirical CDFs (solid line) and fitted normal CDFs (dotted line) for molecular (blue line) and non-molecular (red line) motors with *f* < 350 kPa; *µ* and *σ* in kPa; the two distributions are not significantly different (*p* = 0.20). All comparisons based on Kolmogorov–Smirnov tests.

The distribution of all *f* values is close to lognormal, with log_10_(*f*) following approximately a normal distribution of mean *µ* = 5.07 (corresponding to 159 kPa), the largest measured tension (in a pilus) being 1900 kPa (figure 1*a*). Since the slope of the distribution changes rapidly for *f* = 350 kPa, we have also plotted the distribution of *f* data smaller than this value (90% of the total), which follow very closely a normal distribution of mean ± s.d. = 161 ± 78 kPa (figure 1*b*). Figure 1*c* compares the tensions *f* of molecular and non-molecular motors, which follow distributions that are not significantly different, close to lognormal for all values and normal for *f* < 350 kPa (figure 1*d*).

Motors developing tensions higher than 350 kPa are found in both microorganisms and large animals. In the former, the only ones are pili. In the latter, 23 of 29 (80%) are whole muscles measured *in vivo* (MV) in crustaceans (claw closers) and insects (jump muscles). We shall return to this point later.

### 3.2. Differences exist depending on motor types, taxonomic groups and functional groups

**Figure 2.**
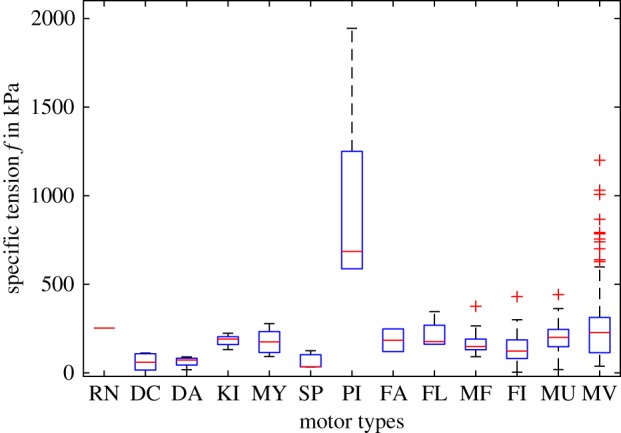
Boxplots of specific tensions per motor type (*n* = 349). The boxes extend from the lower quartile to the upper quartile values with the medians (red line) in between. The whiskers extend to the most extreme data values within 1.5 × IQR. Outliers (red crosses) are tensions beyond the end of the upper whiskers. Motor types: RN, RNA polymerase (*n* = 1); DC, cytoplasmic dynein (4); DA, axonemal dynein (4); KI, kinesin (7); MY, myosin (11); SP, spasmoneme (3); PI, pili (6); FA, *F*_0_/*F*_1_ ATPase (2); FL, flagellum (4); MF, myofibril (16); FI, muscular fibre (97); MU, muscle *in vitro* (89); MV, muscle *in vivo* (105). ANOVA and multiple comparison of means (electronic supplementary material, table S1, motor types with *n* < 5 removed: RN, DC, DA, SP, FA and FL): PI ≠ (KI, MY, MF, FI, MU, MV), FI ≠ MV and MU ≠ MV. Pili PI are significantly different from all other motor types.

Figure 2 shows that the tension for bacterial pili (PI, median 685 kPa, interquartile range (IQR) 663 kPa, *n* = 6) is clearly an outlier with respect to all other motors (median 167 kPa, IQR 134 kPA, *n* = 343). Therefore, in all the following comparisons, pili are excluded.

**Figure 3.**
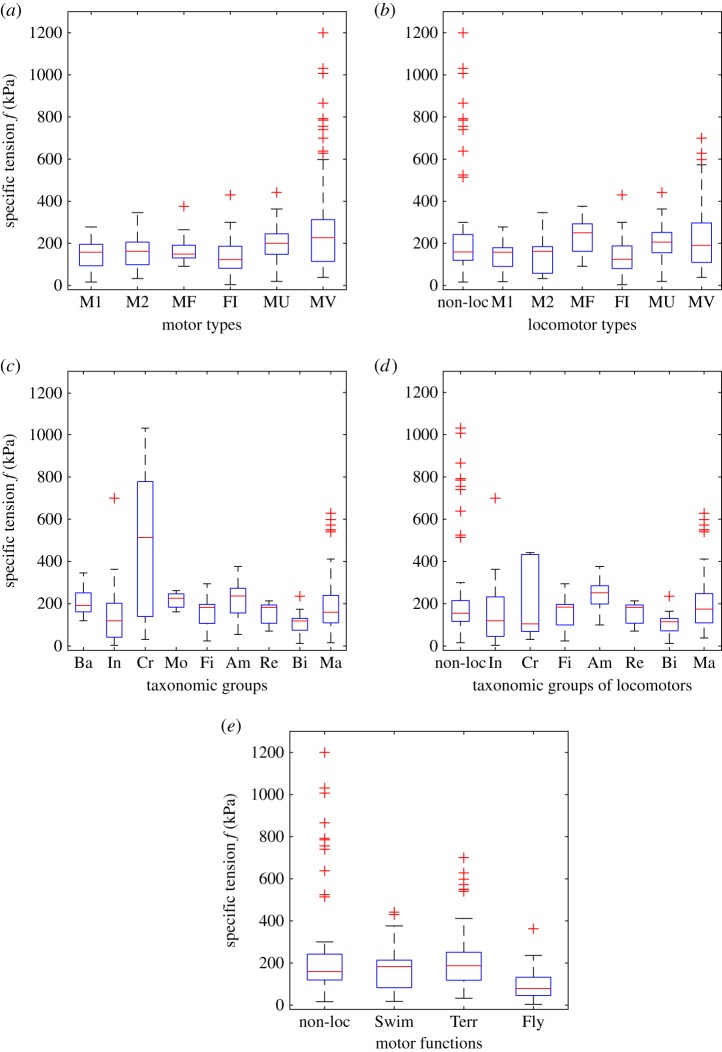
Boxplots of specific tensions of all motors except pili (*n* = 343). Pili were excluded from molecular assemblies (M2), bacteria (Ba) and terrestrial motors (Terr). (*a*) Per motor type. Abbreviations and number of values per class as defined in figure 2, except M1, single molecule (*n* = 27) and M2, molecular assembly (*n* = 9). ANOVA and multiple comparison of means (electronic supplementary material, table S2): MV ≠ (M1, FI, MU). Among the 11 MV outliers, 9 are claw muscles and 2 are jump muscles. (*b*) Same as (*a*) with non-locomotors (non-loc, *n* = 55) as a separate class. ANOVA and multiple comparison of means (electronic supplementary material, table S3): non-loc ≠ (M1, FI) and FI ≠ MV. (*c*) Taxonomic groups: Ba, bacteria (*n* = 7); Pr, protozoa (4); Al, algae (1); Fu, fungi (1); Ec, echinoderms (1); Ar, arachnids (1); In, insects (19); Cr, crustaceans (19); Mo, molluscs (5); Fi, fish (29); Am, amphibian (31); Re, reptiles (5); Bi, birds (18); Ma, mammals (202). Groups with *n* < 5 (protozoa, algae, fungi, echinoderms, arachnids) were removed (remaining data: *n* = 335); ANOVA and multiple comparison of means (electronic supplementary material, table S4): crustaceans are significantly different from all other groups. (*d*) Same as (*c*) for locomotors (*n* = 275) with non-locomotors (*n* = 48) as a separate class. Groups with *n* < 5 were removed (same as in (*c*), plus bacteria and molluscs). Insects (*n* = 17), crustaceans (5), fishes (28), amphibians (25), reptiles (5), birds (17), mammals (178). ANOVA and multiple comparison of means (electronic supplementary material, table S5): non-loc ≠ (Fi, Bi, Ma). (*e*) Per motor function: non-locomotory (*n* = 55), swimming (53), flying (25), terrestrial (210). Abbreviations and number of values per class as given in figure 1*d*, except for Terr (*n* = 210). ANOVA and multiple comparison of means (electronic supplementary material, table S6): non-loc ≠ (Swim, Terr, Fly) and Fly ≠ Terr.

Comparisons of tension without pili per motor types, taxonomic groups and motor functions are shown as boxplots in figure 3 and the corresponding statistical tests (ANOVA and multiple comparison of means) are given in the electronic supplementary material, tables S1–S6. Figure 3*a,b* for motor types indicates that muscles *in vivo* significantly differ from single molecules M1, fibres and muscles *in vitro*, essentially because of the large tensions of non-locomotor muscles. Comparisons of taxonomic groups with number of *f* values greater than or equal to 5 (pili excluded) show that crustaceans differ from all other groups (all motors, figure 3*c*). Finally, comparison of motor functions show that motors used for flight have specific tensions significantly different from those of motors used for moving the organisms on (or with respect to) a solid substrate and non-locomotors differ from all three kinds of locomotors (figure 3*e*).

### 3.3. There is no large-scale variation with cell or body mass

**Figure 4.**
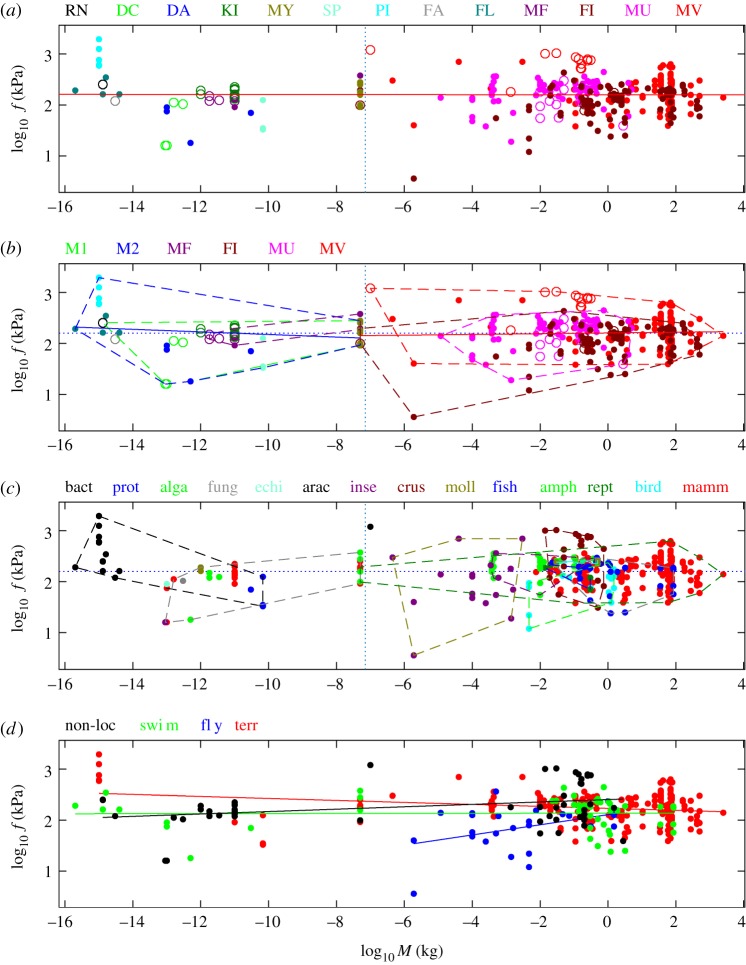
Log–log plot of specific tension versus cell or body mass. (*a*) Locomotors shown as points (*n* = 294) and non-locomotors as circles (*n* = 55). Regression line of all log_10_ *f* versus log_10_ *M* (solid red line, slope −5 × 10^−4^ not significantly different from zero, *p* = 0.90). Regression line of locomotors (slope −6 × 10^−3^ not significantly different from zero, *p* = 0.24) indistinguishable from red line, not shown (see the electronic supplementary material, table S7). Vertical dotted line: mass of cells on the left, of multicellular organisms on the right. Motor types: abbreviations and number of values per type as defined in figure 2. (*b*) Motor types: same abbreviations and numbers as in (*a*), except M1, single molecule (*n* = 27) and M2, molecular assembly (15 with pili). Symbols and colours of points as in (*a*). Points belonging to the same motor type located within the convex polygons shown. Regression lines of molecular motors (M1, M2 and MF, blue line on the left, slope −0.03 not significantly different from zero, *p* = 0.17) and non-molecular motors (FI, MU, MV, red line on the right, slope 7 × 10^−3^ not significantly different from zero, *p* *=* 0.47). For these and other regressions on motor types, see the electronic supplementary material, tables S7–S9. Horizontal dotted blue line is mean log_10_ *f* (kPa) = 2.2. Vertical dotted blue line as in (*a*). (*c*) Taxonomic groups: abbreviations and number of values per class as given in figure 3*c*, except for bacteria (*n* = 13 with pili). On the left side, polygons enclose motors from single cells (black) and from multicellular organisms (grey). For regressions on taxonomic groups, see the electronic supplementary material, tables S10 and S11. Horizontal and vertical dotted lines as in (*b*). (*d*) Motor functions: non-locomotory (*n* = 55), swimming (53), flying (25), terrestrial (216 with pili). Their respective regression lines are shown; their slopes *s* are significantly different from zero (non-loc, *s* = 0.02, *p* = 0.02; Fly, *s* = 0.1, *p* = 0.05; Terr, *s* = −0.02, *p* < 10^−3^) except Swim (*s* = 8 × 10^−4^, *p* = 0.93), see the electronic supplementary material, table S12. In all panels, the scale on the *y*-axis is 1.5 times larger than on the *x*-axis.

Log–log plots of the 329 pairs of (*M*, *f*) values are shown in figure 4. Overall, values of cell and body mass *M* range from 2 × 10^−16^ kg (bacterium) to 2500 kg (elephant), whereas values of specific tension *f* range from 3.6 to 1944 kPa. Hence, whereas *M* varies by more than 19 orders of magnitude, *f* only varies by a factor of 500. For easier reading, polygons enclosing all points of the same category are shown: types of motors (figure 4*b*) and taxonomic groups (figure 4*c*).

Overall, there is no large-scale variation with cell or body mass. Indeed, the power law regression calculated for the entire dataset is *f* = 159 *M^α^* with *α* = −0.5 × 10^−3^ ± 7.7 × 10^−3^ (95% confidence limits −8.2 × 10^−3^, 7.2 × 10^−3^), this slope is not significantly different from zero (*p* = 0.90, figure 4*a*). The slope is not either different from zero for data restricted to molecular motors (M1, M2 and MF, *f* = 83 *M^α^* with *α* = −0.025 ± 0.037, *p* = 0.17, figure 4*b* on the left) and non-molecular motors (FI, MU, MV, *f* = 159 *M^α^* with *α* = 0.0073 ± 0.020, *p* = 0.47, figure 4*b* on the right). Complete description and test of these global regressions are given in the electronic supplementary material, table S7.

We also looked for ‘local’ trends based on the different categories defined previously. For motor types, some slight positive and negative slopes of the regression lines *f* versus *M* were found (electronic supplementary material, tables S8 and S9). For taxonomic groups (electronic supplementary material, tables S10 and S11) and motor functions (electronic supplementary material, table S12), either the slope is not significantly different from zero (according to the *F*-test at level 1%), or the slope is smaller or equal to 0.02 in absolute value.

## 4. Discussion

We discuss in order the choice of specific tension for normalizing forces developed by widely different motors, the similarity of specific tension in molecular and non-molecular motors, the factors explaining the variability of tension, especially in muscles, and the relationship between tension invariance and force–mass scaling.

### 4.1. Specific tension as a size-independent measure of force

In order to compare forces developed by biological motors as different as molecules and muscles, whose spatial scale varies by nearly 7 orders of magnitude and whose applied force varies by nearly 14 orders of magnitude, it is useful to express them in relative values. Because most non-molecular motor forces *F* (FI, MU, MV) are expressed as specific tension (*F*/*A*) in the literature, it is natural to try to express molecular motors similarly.

As *F*/*A* is not available for molecular motors, in order to avoid bias, we defined the cross-section *A* in the most basic way, i.e. from the volume *V* as *A* = *V*^2/3^, which holds for a cube and still holds in order of magnitude for shapes of moderate elongation. This is in line with results of Marden & Allen [18] who found *F* proportional to motor mass *m*^2/3^ for a class of molecular motors, and to the fact that these forces depend on chemical bonds (mainly hydrogen bonds), whose number acting in parallel is expected to depend on the cross section. For defining the cross-section, we were extremely careful to select the acting part of the motor (ignoring the ‘passive’ tails) so that the shape was of moderate elongation. For example to estimate the volume of the myosin motor, we only considered the heads and ignored the tail which does not contribute to the actin–myosin interaction. We will return to this topic in the last subsection ‘Scaling with motor's mass’ and suggest below an order-of-magnitude interpretation.

### 4.2. Invariance of specific tension in molecular and non-molecular motors

The main characteristics found here for the values of tension *f* in both molecular (M1, M2, MF) and non-molecular motors (FI, MU, MV) are (table 5): (i) their almost equal median tensions (approx. 170 kPa), (ii) their similar ranges of variation (60 < *f* < 350 kPa for 90% of motors), and (iii) the approximately five times higher tensions exerted by pili (600 < *f* < 2000 kPa). These three characteristics can be understood from basic physical considerations.

#### 4.2.1. Molecular motors

Molecular motors are proteins that produce mechanical energy by changing their three-dimensional conformation. They move in steps whose length is of the order of magnitude of their size *a*_0_, which is typically *a*_0_ ∼ 6 nm [195,196]. The steps are mainly powered by ATP with free energy *W*_0_ ≃ 12*kT* ≃ 0.5 × 10^−19^ J/molecule at *T* = 300 K [197]. Therefore, the elementary force *F*_0_ developed by motor proteins is of order of magnitude *F*_0_ ∼ *W*_0_/*a*_0_ ∼ 8 pN and the corresponding force per unit cross-sectional area *f* is *f* ∼ *F*_0_/*a*_0_^2^ ≃ *W*_0_/*a*_0_^3^ ∼ 200 kPa. This is close to the average value found for molecular motors (M1, M2 and MF, table 5). This order-of-magnitude estimate is based on a perfect transduction of chemical into mechanical energy. Taking into account the actual efficiency would not change this order of magnitude since molecular motors are known to have a high efficiency—often exceeding 50% (e.g. [198,199]), in particular, 80–95% for kinesin [197] and up to 100% for F1-ATPase [8].

Molecular motors, like other proteins, owe their properties to a three-dimensional structure mainly held by H-bonds and other weak forces [200,201]. In order to act near (but not at) thermal equilibrium and not to break the motor protein, the elementary motor force should not exceed *kT* divided by the distance over which H-bonds operate, i.e. the size of the water molecule, $a_{H_{2}O}≃0.3\;\mathrm{nm}$. This yields the minimum size, $a_{0}>a_{H_{2}O}\times (W_{0}/kT)≃4\;\mathrm{nm}$, and maximum tension, *f* ≃ *W*_0_/*a*_0_^3^ < 800 kPa, of molecular motors. This order of magnitude estimate is similar to the maximum tension observed in molecular motors (table 5) with the notable exception of pili.

Pili, which are virtually universal in prokaryotes [202], have exceptional mechanical properties of stretching and adhesion, and some of them can withstand extreme forces, with an important role played by covalent bonds (e.g. [203]) so that the above order-of-magnitude estimate, based on weak forces, does not apply to them. In order to compare pili with other structures, we have only considered steady-state unwinding forces (e.g. [60]). Even then, pili can still reach extreme specific tensions, with a median four times higher than that of other motors.

#### 4.2.2. Non-molecular motors

The most striking result of this paper is that the formally defined tension of molecular motors turns out to be similar to the value *f* ≃ 200 kPa typical of muscle fibres. A hint to this uniformity stems from the basic arrangement of myosin motors in striated muscles (reviewed in e.g. [13,204]). Most of the space within muscle fibres is occupied by protein thick filaments along which groups of myosin globular motors (heads) are protruding with an axial spacing *e* = 14.6 nm. These motors are cyclically attaching to (and detaching from) adjacent thin filaments of actin to form the cross-bridges, and enable thin and thick filaments to slide past each other. Along each half thick filament (of total length 2*l* ≃ 1.6 µm, neglecting for this order-of-magnitude estimate a bare zone of smaller length free of motors) about 150 myosin molecules exert forces that add in parallel and only about one-third of the cross-bridges are attached during isometric contraction [47,205]. Therefore, the number of active individual myosin motors along each half thick filament is *N* ≃ 50. (Note that since *l/e* ≃ 50, this might imply that only one motor per group of three can attach simultaneously, a likely consequence of steric constraints brought about by the three-dimensional structure enabling transitory conformational changes.) With *N* motors acting in parallel each exerting a force *F*_myosin_ the total force per thick filament is *NF*_myosin_. Each thick filament and its associated lattice of thin filaments occupies an equivalent cross-section *s* ≃ *d*^2^, where *d* ≃ 40 nm is the lateral spacing of thick filaments, so the total tension in the structure is *f*_fibre_ ≃ *NF*_myosin_/*s* which acts (in series) along the length of the fibre. Tables 3 and 4 show that the myosin motor, of equivalent cross-sectional area *A* ≃ 36 nm^2^, exerts a mean force *F*_myosin_ ≃ *f*_myosin_*A* ≃ 7 pN. Substituting the values of *F*_myosin_, *N* and *s* in the above formula yields the tension in the structure *f*_fibre_ ≃ 240 kPa.

This rough estimate enables us to understand why the tension of muscles (≃*f*_fibre_) is of the same order of magnitude as the tension of the myosin motor *f*_myosin_ ≃ 190 kPa. Indeed, the tensions of muscle fibres and of myosin motors are in the ratio *f*_fibre_/*f*_myosin_ ≃ *NA/s*, and the myosin motors are arranged so that the number *N* of them acting simultaneously in parallel is approximately equal to the ratio *s/A* of the equivalent cross-sectional area of each thick–thin filament structure to that of an individual myosin motor head, which is not surprising because of steric constraints.

### 4.3. Origins of variability of specific tension in various motors

Overall, tensions in most molecular and non-molecular motors are distributed around their means according to similar Gaussian functions with coefficients of variation s.d./mean ≃ 0.5. This variability may arise from methodological, experimental and biological factors.

#### 4.3.1. Methodological and experimental factors

The cross-section *A* of molecular motors was estimated from their mass *m* using the formulae *A* = *V*^2/3^ and *V* = *m*/*ρ* with protein density *ρ* ≃ 10^−3^ pg nm^−3^. This is admittedly rough, since the longer dimension of the motors considered can differ from the cross-diameter by nearly a factor of 2. The resulting error may not be negligible compared with the observed variability of specific tension in molecular motors, in which more than 80% of *f* values are within one-third of the median and twice the median (see Q10, Q90 and median in table 5, second line).

Although we did not have to estimate the cross-section for muscles, their tensions show the same variability on *f* as molecular motors (Q10 is one-third the median and Q90 twice the median, see table 5, third line). Their cross-sectional area has sometimes been corrected for the area occupied by mitochondria (dragonfly, [116]), sometimes not (beetle, [115]) and never for the sarcoplasmic reticulum (e.g. [206]). The pennation angle has not always been taken into account. Temperature during the experiments has been noted and is usually close to the working temperature of the muscle. Although data are not fully homogeneous, the similarity of the distributions of specific tensions measured *in vivo* and *in vitro* suggests that uncorrected factors do not introduce important bias. In principle, corrections for these factors should lead to less variable data. However, no corrections have been attempted for two reasons. First, the information needed is not always provided, so corrections cannot be done systematically. Second, these corrections would probably have no incidence on the qualitative conclusions, and might even be less convincing than unmodified data.

Isometric tension in single skeletal muscle fibres (FI) is approximately 35% smaller than in whole muscles (MU or MV) (figure 3*a*). This difference probably results from the experimental conditions, most measurements of single fibres being performed after chemical or mechanical skinning. It produces swelling of the fibres and reduces the specific tension. Median tension is about the same for whole muscles when measured *in vitro* (MU, 200 kPa) and *in vivo* (MV, 227 kPa) (figure 3*a*,*b*). This indicates that the tension for muscles in behaving animals is close to the maximum they can develop in *in vitro* conditions.

It must also be realized that detailed physiologically and ecologically relevant comparisons between similar motors in different taxonomic groups are hindered by their unequal levels of investigation; for example, muscles MU have been studied in 29 vertebrate species, but only 13 invertebrate species (table 4).

#### 4.3.2. Biological factors

Further sources of variability are probably biological. At the molecular level, variability stems from differences within and across families of single motor proteins (M1). At the supramolecular level, notably in propulsion organelles and muscles, elementary molecular forces are expressed via an organization that introduces further variations and specific adaptations to the diversity of mechanical problems they had to solve. More factors being involved, the values of their tension is *a priori* less easy to predict, explaining the variability observed. Nonetheless, as shown in figure 3*a*, after removal of pili, the variability of specific tension between the different types of molecular motors studied is larger in motors M1 and M2 than in myofibrils. The structural and functional homogeneity of myofibrils contrasts with the heterogeneity of the other molecular motors.

Neglecting experimental errors and pili being set aside, tensions of non-molecular motors (FI, MU, MV) vary approximately in the same range as tensions of molecular motors (M1, M2 and MF) with the same statistical distribution (figure 1*c*,*d*). So, notwithstanding their myosin-based molecular homogeneity, the diversity in geometry and adaptation of muscular motors leads to variations in tension equivalent to those resulting from the diversity of molecules and their arrangements in molecular motors. It is remarkable that so many different mechanisms lead to the same final distributions of force per cross-sectional area at the microscopic and macroscopic levels.

### 4.4. Variability of tensions in whole muscles

The variability of tension in muscles has been the subject of thorough research. An important adaptive factor is sarcomere length. As predicted by the sliding filament model of muscle contraction, long filaments and long overlap between thick and thin filaments should occur in fibres with long sarcomeres. As in long overlap zones more actin–myosin cross-bridges should be formed, the maximum tension which a fibre can produce should be correlated with sarcomere length [207,208]. The resting sarcomere length exhibits little variation in insect and vertebrate muscles (2–4 µm), but much greater variations in crustacean muscles (7–17 µm). Overall, tension scales isometrically with the resting sarcomere length [157]. In particular, the claw closer muscles of cancer crabs exhibit both the longest sarcomere lengths and extreme mean crushing forces (525–1030 kPa; table 4 and figure 3*c*). This is a special adaptation of shell-crushing non-locomotory motors which is not found in locomotors (figure 3*d*).

Many other factors have been invoked to explain the variations in muscle tension, such as the density of the myosin filaments, the non-uniformity of sarcomere length along the fibres, the diameter of myofibrillar bundles, the actin : myosin filament ratios and the cross-bridge duty factors. For example, the slightly higher tension than in other groups found in amphibians and molluscs (except crustaceans; figure 3*c*) may be explained by their higher proportion of fast oxidative fibres and their higher relative myofibrillar volume [4,206]. However, these various factors apparently play a minor role in arthropod and vertebrate muscles as more than 80% of the variation in muscle tension in a series of muscles from these groups can be explained by the resting sarcomere length ([157] and references therein).

Two characteristics other than tension contribute to muscle performance: speed of contraction (and relaxation) and endurance. They influence tension because high tension requires that most of the cross-sectional area of a fibre be myofibrils, whereas high endurance requires a large mitochondrial volume and short twitch duration requires an extended sarcoplasmic reticulum. Therefore, trade-offs are inherent in the functional design of muscles so that a muscle cannot be simultaneously strong, enduring and rapid. This is the reason why rapid muscles are weak (either enduring, e.g. katytid singing muscles, or not, e.g. lobster sound-producing muscles with their hypertrophied SR) [208]. However, special adaptations in the oscillatory (asynchronous) flight muscles of insects result in high contraction frequencies without a large volume of SR, which leaves room for more mitochondria, but their strength is nevertheless limited by the endurance requirements of flight [208]. They are built optimally for maximum output of energy in their narrow contraction range, whereas most vertebrate sarcomeres are optimized for optimal mechanical conversion of chemical energy across a wider contraction range [209]. These different adaptions contribute to the variability observed. Overall, the similarity of muscle tensions is essentially owing to the similarity of fibre structure and thick filament length across muscles and species, in contrast with the variability of muscle speeds which are affected by the variability of thin filament lengths (e.g. [210]).

It is remarkable that tension is smaller in flight locomotors (median 79 kPa) than in terrestrial locomotors (median 187 kPa) and in swim locomotors (183 kPa), although only the difference for terrestrial locomotors is significant according to ANOVA at level 5% (figure 3*e*). Despite the high power needed for flight, the high frequencies required may impose a large concentration of mitochondria and, at least in birds, of sarcoplasmic reticulum at the expense of myofibrils. Solving this issue will need further investigation.

### 4.5. Absence of large-scale trend with cell's or body's mass

Given the constancy in both central value (mean or median) and dispersion (s.d. or interquartile range) of *f* in molecular and non-molecular motors, it is not surprising that the regressions in a log–log plot of *f* against *M*, the mass of the cell (for subcellular motors) or body (for cellular and supracellular motors) from which the motor is extracted, give no evidence of overall trend (figure 4*a*,*b*). Other variables for the mass might be used, but their implementation is difficult because they are often ill-defined or unknown. This is the reason why we chose for the horizontal axis a proxy of the mass that the motor moves—the mass of the next higher hierarchical level, i.e. the cell's mass for subcellular motors (M1, M2, MF) and the animal's mass for cellular and supracellular motors (FI, MU, MS). This definition is simple, unambiguous, known in almost all cases and discriminant with a range extending over 18 orders of magnitude. If we had chosen the motor's mass *m* for the horizontal axis, the range would have been still wider since the minimum mass would be 10^−22^ kg (kinesin) and the maximum mass > 1 kg (muscle), so that as the overall range of *f* would remain the same, the slope of the regression line would become still closer to zero.

The absence of global trends does not preclude the existence of ‘local’ trends, i.e. regression lines with slope significantly different from zero, for specific classes of motors extending on a narrower mass range. Several examples of such significant trends were found (see the electronic supplementary material, tables S8–S12) but their slopes are small and difficult to interpret. These small-scale relationships are outside the scope of this paper which focuses on a large-scale study. The wide range of size, mass and area considered allows one to transcend the possible variations specific to certain categories.

### 4.6. Scaling with motor's mass

A different approach based on force *F* and motor mass *m* strengthens this conclusion. Indeed, Marden & Allen [18] studied the scaling of forces with motor's mass for two classes of animal- and human-made motors and found that one of them, ‘Group 1’ motors, producing translational motion, scale allometrically with motor mass *m*, as *F* ≃ 10^3^*m*^2/3^ (with *F* in Newtons and *m* in kilograms). We show below that this scaling, expressed in terms of specific tension *f*, is in good agreement with the typical specific tension found in the present paper (approx. 200 kPa). Consider first the order-of-magnitude approximation of cubes of section *A*. With the mass density *ρ* ≃ 10^3^ kg m^−3^, the motor mass is *m* ≃ *ρA*^3/2^, so that the scaling above *F* ≃ 10^3^ (*ρA*^3/2^)^2/3^ yields the tension *f* = *F/A* ≃ 10^3^ *ρ*^2/3^ ≃ 100 kPa. This is a minimum value since replacing the cubic approximation by an elongated shape, with a ratio length/width *r*, with width *d* ≃ *A*^1/2^, would yield *m* ≃ *rρA*^3/2^, whence *f* ≃ 100 *r*^2/3^ kPa. Thus, the mass–force scaling for Group 1 motors found by Marden & Allen [18] implies the constancy of their specific tension with a constant value consistent with that found here.

The above argument might also explain why three ‘molecular motors’ corresponding in part to our ‘M2 motors’ (bacterial flagellum, mammalian flagellum and spasmoneme) are shifted to the right of the fitted line (see red circles in fig. 1 of [18]). Indeed, the mass *m* considered is the mass of the whole organelle, whose length far exceeds the square root of the section (i.e. r≫1). This implies that *m* is much larger than *ρA*^3/2^, so that a constant value of *f* yields a smaller value of *F*/*m*^2/3^.

However, for the other group of motors (Group 2) defined by Marden & Allen [18], the biological motor forces are generally deduced from the motion of the whole organism against gravity, which implies various joints and lever arms connecting the motor to the organism. It is, therefore, difficult to compare these data with those considered in this paper, which are directly measured at the level of the muscle (or of the fibre or the molecular motor).

## 5. Concluding remarks

The main result of this paper is that, despite their diversity, molecular and macroscopic biological motors do exert similar forces per unit cross-sectional area, which enables us to unify biological motors of different sizes and varied functions, from the motion of animals and microorganisms to cargo transport in cells or DNA transcription. The similarity of tensions of macroscopic muscles and fibres is not surprising as it stems from the similarity of fibres' basic architecture. In turn, the similarity of the tensions of molecular motors is owing to the basic physical properties of protein machines, and we have given an order-of-magnitude estimate of this tension from basic physics. Finally, we have shown that the tension in muscle fibres is similar to that of the myosin motor in particular because of the arrangement of these motors in the fibres, owing to steric constraints.

The approximate constancy of the maximum force per unit area *f* found in this paper from molecules to muscles implies general scaling laws for the motion of organisms [211] and raises the question of relating these laws to basic biological and physical constraints. Moreover, it calls for an explanation of why human-engineered motors, which are not based on ATP hydrolysis and hydrogen bond forces, show very similar specific tension to biological motors [18,19].

## Supplementary Material

Supplementary Tables

## Data accessibility

All supporting data are made available in tables 2–5 and the electronic supplementary material, tables S1–S12.
