# Supplementary material for: Force per cross-sectional area from molecules to muscles: a general property of biological motors
Source: R Soc Open Sci. 2016 Jul 20;3(7):160313. doi: 10.1098/rsos.160313 (PMC4968477; doi:10.1098/rsos.160313)
Supplement: Supplementary Tables [file rsos160313supp1.docx]

SUPPLEMENTARY TABLES

FORCE PER CROSS-SECTIONAL AREA FROM MOLECULES TO MUSCLES:

A GENERAL PROPERTY OF BIOLOGICAL MOTORS

Jean-Pierre Rospars and Nicole Meyer-Vernet

Content

1. Tables S1 to S6: ANOVA and multiple comparison of means

Tables S1: Comparison of detailed motor types

Tables S2: Comparison of grouped motor types

Tables S3: Comparison of locomotor types

Tables S4: Comparison of taxonomic groups

Tables S5: Comparison of taxonomic groups for locomotors

Tables S6: Comparison of motor functions

2. Tables S7 to S12: log-log regressions of specific tension *vs*. cell or body mass

Table S7: General regressions

Table S8: Motor types

Table S9: Locomotor types

Table S10: Taxonomic groups

Table S11: Taxonomic groups for locomotors

Table S12: Motor functions

1. TABLES S1 TO S6: ANOVA AND MULTIPLE COMPARISON OF MEANS

Note: In all comparisons, groups with *n* < 5 were excluded. In all Tables except S1a-c, pili were excluded.

Legend common to Tables S1c, S1f, S2c, S3c etc.

Lower: lower bound of the difference of means.

Estimate: estimated difference of means.

Upper: upper bound of the difference of means.

P: *P*-value. Critical value based on Tukey-Kramer adjustment method.

Test: if P > 0.05, means are not significantly different (=), otherwise difference is significant (≠).

1.1. Comparisons of detailed motor types (Fig. 2)

Table S1a. Anova of specific tensions per motor type

Variation Sum of squares df Mean square F_obs_ F_th_ P

----------------------------------------------------------------

Between groups 4447559.80 6 741259.97 27.71 2.13 10^-26^

Within groups 8666552.00 324 26748.62

Total variation 13114111.80 330

----------------------------------------------------------------

Table S1b. Mean and SEM of specific tensions per motor type

n Mean SEM

-------------------

KI 7 181.57 61.82

MY 11 174.55 49.31

PI 6 956.33 66.77

MF 16 172.56 40.89

FI 97 135.65 16.61

MU 89 195.37 17.34

MV 105 280.55 15.96

-------------------

Table S1c. Multiple comparison of means for motor type

Comparison Lower Estimate Upper P Test

------------------------------------------------

KI - MY -226.11 7.03 240.17 1.00 =

KI - PI -1043.03 -774.76 -506.49 4×10^-8^  ≠

KI - MF -209.51 9.01 227.52 1.00 =

KI - FI -142.79 45.93 234.64 0.99 =

KI - MU -203.08 -13.80 175.49 1.00 =

KI - MV -287.21 -98.98 89.25 0.71 =

MY - PI -1026.51 -781.79 -537.06 4×10^-8^  ≠

MY - MF -186.88 1.98 190.85 1.00 =

MY - FI -114.51 38.90 192.31 0.99 =

MY - MU -174.94 -20.83 133.29 1.00 =

MY - MV -258.82 -106.01 46.81 0.39 =

PI - MF 552.94 783.77 1014.61 4×10^-8^  ≠

PI - FI 617.83 820.69 1023.54 4×10^-8^  ≠

PI - MU 557.58 760.96 964.35 4×10^-8^  ≠

PI - MV 473.38 675.78 878.18 4×10^-8^  ≠

MF - FI -93.20 36.92 167.03 0.98 =

MF - MU -153.75 -22.81 108.13 1.00 =

MF - MV -237.40 -107.99 21.42 0.17 =

FI - MU -130.50 -59.73 11.05 0.16 =

FI - MV -212.81 -144.91 -77.00 4×10^-8^  ≠

MU - MV -154.66 -85.18 -15.71 6×10^-8^  ≠

------------------------------------------------

1.2. Comparison of grouped motor types (Fig. 3A)

Note: pili excluded from M2.

Table S2a. Anova of specific tensions per motor type

Variation Sum of squares df Mean square F_obs_ F_th_ P

-----------------------------------------------------------------

Between groups 1190555.26 5 238111.05 10.92 2.24 10^-9^

Within groups 7347461.78 337 21802.56

Total variation 8538017.04 342

-----------------------------------------------------------------

Table S2b. Mean and SEM of specific tensions per motor type

n Mean SEM

-------------------

M1 27 146.11 28.42

M2 9 158.11 49.22

MF 16 172.56 36.91

FI 97 135.65 14.99

MU 89 195.37 15.65

MV 105 280.55 14.41

-------------------

Table S2c. Multiple comparison of means per motor type

Comparison Lower Estimate Upper P Test

----------------------------------------------

M1 - M2 -173.96 -12 149.96 1.00 =

M1 - MF -159.20 -26.45 106.30 0.99 =

M1 - FI -81.09 10.47 102.02 1.00 =

M1 - MU -141.71 -49.26 43.19 0.65 =

M1 - MV -225.24 -134.44 -43.65 4×10^-4^  ≠

M2 - MF -189.78 -14.45 160.87 1.00 =

M2 - FI -124.16 22.47 169.09 1.00 =

M2 - MU -184.44 -37.26 109.92 0.98 =

M2 - MV -268.59 -122.44 23.71 0.16 =

MF - FI -76.62 36.92 150.46 0.94 =

MF - MU -137.07 -22.81 91.45 0.99 =

MF - MV -220.92 -107.99 4.94 0.07 =

FI - MU -121.49 -59.73 2.04 0.06 =

FI - MV -204.17 -144.91 -85.65 2×10^-8^ ≠

MU - MV -145.81 -85.18 -24.55 9×10^-4^ ≠

----------------------------------------------

1.3. Comparison of locomotor types (Fig. 3B)

Note: pili excluded from M2.

Table S3a. Anova of specific tensions per locomotor type

Variation Sum of squares df Mean square F_obs_ F_th_ P

------------------------------------------------------------

Between groups 787641.18 6 131273.53 5.69 2.13 10^-5^

Within groups 7750375.86 336 23066.59

Total variation 8538017.04 342

------------------------------------------------------------

Table S3b. Mean and SEM of specific tensions per locomotor type

n Mean SEM

-----------------------

Non-loc 55 275.49 20.48

M1 15 144.87 39.21

M2 7 150.71 57.40

MF 5 233.60 67.92

FI 92 136.68 15.83

MU 76 205.08 17.42

MV 93 219.61 15.75

-----------------------

Table S3c. Multiple comparison of means per locomotor type

Comparison Lower Estimate Upper P Test

------------------------------------------------

Non-loc - M1 0.19 130.62 261.06 0.05 ≠

Non-loc - M2 -54.92 124.78 304.47 0.38 =

Non-loc - MF -167.27 41.89 251.05 1.00 =

Non-loc - FI 62.49 138.81 215.13 2×10^-6^  ≠

Non-loc - MU -8.86 70.41 149.68 0.12 =

Non-loc - MV -20.29 55.88 132.05 0.32 =

M1 - M2 -210.81 -5.85 199.12 1.00 =

M1 - MF -319.97 -88.73 142.50 0.92 =

M1 - FI -116.50 8.19 132.87 1.00 =

M1 - MU -186.73 -60.21 66.30 0.80 =

M1 - MV -199.34 -74.75 49.85 0.57 =

M2 - MF -345.08 -82.89 179.31 0.97 =

M2 - FI -161.53 14.03 189.60 1.00 =

M2 - MU -231.23 -54.36 122.50 0.97 =

M2 - MV -244.40 -68.90 106.60 0.91 =

MF - FI -108.70 96.92 302.54 0.81 =

MF - MU -178.22 28.52 235.26 1.00 =

MF - MV -191.58 13.99 219.55 1.00 =

FI - MU -137.81 -68.40 1.01 0.06 =

FI - MV -148.78 -82.93 -17.09 4×10^-3^  ≠

MU - MV -83.77 -14.53 54.71 1.00 =

------------------------------------------------

1.4. Comparison of taxonomic groups (Fig. 3C)

Note: pili excluded from Bact.

Table S4a. Anova of specific tensions per taxonomic group

Variation Sum of squares df Mean square F_obs_ F_th_ P

----------------------------------------------------------------

Between groups 1958630.00 8 244828.75 14.65 1.97 3×10^-18^

Within groups 5447066.76 326 16708.79

Total variation 7405696.76 334

----------------------------------------------------------------

Table S4b. Mean and SEM of specific tension per taxonomic group

n Mean SEM

---------------------

Bact 7 211.86 48.86

Inse 19 174.87 29.65

Crus 19 495.79 29.65

Moll 5 216.60 57.81

Fish 29 157.59 24.00

Amph 31 218.10 23.22

Rept 5 155.20 57.81

Bird 18 106.50 30.47

Mamm 202 183.79 9.09

---------------------

Table S4c. Multiple comparison of means per taxonomic group

Comparison Lower Estimate Upper P Test

------------------------------------------------

Bact - Inse -140.29 36.98 214.25 1.00 =

Bact - Crus -461.20 -283.93 -106.66 2×10^-5^  ≠

Bact - Moll -239.51 -4.74 230.02 1.00 =

Bact - Fish -114.57 54.27 223.11 0.99 =

Bact - Amph -174.02 -6.24 161.54 1.00 =

Bact - Rept -178.11 56.66 291.42 1.00 =

Bact - Bird -73.23 105.36 283.95 0.66 =

Bact - Mamm -126.07 28.07 182.21 1.00 =

Inse - Crus -451.00 -320.92 -190.83 9×10^-8^  ≠

Inse - Moll -243.25 -41.73 159.79 1.00 =

Inse - Fish -101.05 17.29 135.62 1.00 =

Inse - Amph -160.04 -43.22 73.59 0.97 =

Inse - Rept -181.85 19.67 221.19 1.00 =

Inse - Bird -63.50 68.37 200.25 0.80 =

Inse - Mamm -105.12 -8.91 87.30 1.00 =

Crus - Moll 77.67 279.19 480.71 6×10^-4^  ≠

Crus - Fish 219.87 338.20 456.54 9×10^-8^  ≠

Crus - Amph 160.88 277.69 394.51 9×10^-8^  ≠

Crus - Rept 139.07 340.59 542.11 6×10^-6^  ≠

Crus - Bird 257.41 389.29 521.17 9×10^-8^  ≠

Crus - Mamm 215.79 312.00 408.21 9×10^-8^  ≠

Moll - Fish -135.13 59.01 253.16 0.99 =

Moll - Amph -194.72 -1.50 191.73 1.00 =

Moll - Rept -192.18 61.40 314.98 1.00 =

Moll - Bird -92.58 110.10 312.78 0.76 =

Moll - Mamm -148.70 32.81 214.32 1.00 =

Fish - Amph -164.09 -60.51 43.07 0.67 =

Fish - Rept -191.76 2.39 196.53 1.00 =

Fish - Bird -69.22 51.09 171.39 0.93 =

Fish - Mamm -105.82 -26.20 53.42 0.98 =

Amph - Rept -130.33 62.90 256.12 0.99 =

Amph - Bird -7.21 111.60 230.41 0.09 =

Amph - Mamm -43.03 34.31 111.65 0.91 =

Rept - Bird -153.98 48.70 251.38 1.00 =

Rept - Mamm -210.10 -28.59 152.92 1.00 =

Bird - Mamm -175.91 -77.29 21.34 0.27 =

------------------------------------------------

1.5. Comparison of taxonomic groups for locomotors (Fig. 3D)

Note: pili excluded from Bact.

Table S5a. Anova of specific tensions per taxonomic group

Variation Sum of squares df Mean square F_obs_ F_th_ P

--------------------------------------------------------------

Between groups 507666.79 7 72523.83 3.33 2.04 2×10^-3^

Within groups 6853764.52 315 21757.98

Total variation 7361431.31 322

--------------------------------------------------------------

Table S5b. Mean and SEM of specific tensions per taxonomic group

n Mean SEM

------------------------

Non-loc 48 268.21 21.29

Inse 17 183.92 35.78

Crus 5 218.00 65.97

Fish 28 159.11 27.88

Amph 25 241.52 29.50

Bird 17 102.53 35.78

Rept 5 155.20 65.97

Mamm 178 189.83 11.06

------------------------

Table S5c. Multiple comparison of means per taxonomic group

Comparison Lower Estimate Upper P Test

--------------------------------------------------

Non-loc - Inse -41.89 84.29 210.47 0.46 =

Non-loc - Crus -159.88 50.21 260.30 1.00 =

Non-loc - Fish 2.79 109.10 215.41 0.04 ≠

Non-loc - Amph -83.58 26.69 136.96 1.00 =

Non-loc - Rept -97.08 113.01 323.10 0.73 =

Non-loc - Bird 39.50 165.68 291.86 2×10^-3^  ≠

Non-loc - Mamm 5.67 78.38 151.09 0.02 ≠

Inse - Crus -261.53 -34.08 193.36 1.00 =

Inse - Fish -112.65 24.81 162.27 1.00 =

Inse - Amph -198.14 -57.60 82.94 0.92 =

Inse - Rept -198.73 28.72 256.16 1.00 =

Inse - Bird -71.96 81.39 234.73 0.75 =

Inse - Mamm -119.40 -5.91 107.58 1.00 =

Crus - Fish -158.16 58.89 275.95 0.99 =

Crus - Amph -242.54 -23.52 195.50 1.00 =

Crus - Rept -219.95 62.80 345.55 1.00 =

Crus - Bird -111.98 115.47 342.92 0.79 =

Crus - Mamm -174.55 28.17 230.90 1.00 =

Fish - Amph -205.43 -82.41 40.60 0.46 =

Fish - Rept -213.15 3.91 220.96 1.00 =

Fish - Bird -80.88 56.58 194.04 0.92 =

Fish - Mamm -121.61 -30.72 60.17 0.97 =

Amph - Rept -132.70 86.32 305.34 0.93 =

Amph - Bird -1.55 138.99 279.53 0.06 =

Amph - Mamm -43.79 51.69 147.18 0.73 =

Rept - Bird -174.78 52.67 280.12 1.00 =

Rept - Mamm -237.35 -34.63 168.10 1.00 =

Bird - Mamm -200.79 -87.30 26.19 0.28 =

--------------------------------------------------

1.6. Comparison of motor functions (Fig. 3E)

Note: pili excluded from Terr.

Table S6a. Anova of specific tensions per motor function

Variation Sum of squares df Mean square F_obs_ F_th_ P

-------------------------------------------------------------

Between groups 613750.60 3 204583.53 8.75 2.63 10^-5^

Within groups 7924266.44 339 23375.42

Total variation 8538017.04 342

-------------------------------------------------------------

Table S6b. Mean and SEM of specific tensions per motor function

n Mean SEM

------------------------

Non-loc 55 275.49 20.62

Swim 53 168.74 21.00

Terr 210 197.75 10.55

Fly 25 100.38 30.58

------------------------

Table S6c. Multiple comparison of means per motor function

Comparison Lower Estimate Upper P Test

-------------------------------------------------

Non-loc - Swim 31.15 106.76 182.36 2×10^-3^ ≠

Non-loc - Terr 18.24 77.74 137.23 4×10^-3^  ≠

Non-loc - Fly 80.36 175.11 269.85 10^-5^  ≠

Swim - Terr -89.39 -29.02 31.36 0.60 =

Swim - Fly -26.95 68.35 163.65 0.25 =

Terr - Fly 14.27 97.37 180.47 0.01 ≠

-------------------------------------------------

2. TABLES S7 TO S12: LOG-LOG REGRESSIONS OF SPECIFIC TENSION *VS*. CELL OR BODY MASS

Legend common to Tables S7 to S12

Group: names abbreviated as in Fig. 4. Pili (PI) included in M2, Bact and Terr. Pili excluded in M2NoPI, BactNoPI, TerrNoPI.

n: number of points (groups with n < 5 were excluded).

x_bar_: mean of *x* = log_10_(*M*) for the specified group.

y_bar_: mean of log_10_(*f*) for the specified group.

inter: intercept of regression line with log_10_(*f*) axis at *M* = 1 kg.

slope: slope of regression line, log_10_(*f*) = inter + slope × log_10_(*M*).

r^2^: coefficient of determination (variation explained by the regression).

F_obs_: observed F.

F_th_: theoretical F at level 0.95 with 1 and n – 2 degrees of freedom.

P: P value.

S: if 1, slope of regression line significantly different from 0 (P < 0.01), 0 otherwise.

Table S7. General regressions (Figs. 4A and 4B)

Group n x_bar_  y_bar_  inter slope r^2^ F_obs_ F_th_ P S

-------------------------------------------------------------------

All 349 -1.98 2.20 2.20 -5×10^-4^  4×10^-5^  0.02 3.87 0.90 0

Molecular 58 -11.16 2.20 1.92 -0.03 0.03 1.91 4.01 0.17 0

Non-molec 291 -0.15 2.20 2.20 7×10^-3^  2×10^-3^  0.53 3.87 0.47 0

Locomotor 294 -1.18 2.19 2.18 -6×10^-3^  5×10^-3^  1.39 3.87 0.24 0

Non-loco 55 -6.23 2.26 2.40 0.02 0.10 6.03 4.02 0.02 0

-------------------------------------------------------------------

Table S8. Motor types (Fig. 4B)

Group n x_bar_ y_bar_  inter slope r^2^ F_obs_ F_th_ P S

------------------------------------------------------------

M1 27 -10.16 2.07 2.62 0.05 0.16 4.76 4.24 0.04 0

M2 42 -11.53 2.20 1.75 -0.04 0.07 2.98 4.08 0.09 0

M2NoPI 9 -13.30 2.10 0.51 -0.12 0.66 13.40 5.59 8×10^-3^  1

MF 16 -10.20 2.21 2.93 0.07 0.66 26.62 4.60 1×10^-4^  1

FI 97 0.11 2.05 2.05 0.05 0.07 7.02 3.94 9×10^-3^  1

MU 89 -1.50 2.24 2.31 0.05 0.06 5.30 3.95 0.02 0

MV 105 0.76 2.31 2.34 -0.04 0.04 4.74 3.93 0.03 0

------------------------------------------------------------

Table S9. Locomotor types (Fig. 4B)

Group n x_bar_  y_bar_  inter slope r_2_ F_obs_ F_th_ P S

------------------------------------------------------------

M1 15 -8.61 2.08 2.88 0.09 0.49 12.68 4.67 3×10^-3^  1

M2 13 -13.87 2.46 -0.29 -0.20 0.60 16.29 4.84 2×10^-3^  1

M2NoPI 7 -12.90 2.06 0.50 -0.12 0.66 9.81 6.61 0.03 0

MF 5 -8.04 2.32 3.32 0.12 0.78 10.64 10.13 0.05 0

FI 92 0.21 2.05 2.04 0.05 0.08 7.68 3.95 7×10^-3^  1

MU 76 -1.54 2.27 2.36 0.06 0.11 8.84 3.97 4×10^-3^  1

MV 93 1.06 2.24 2.23 0.01 4×10^-3^ 0.35 3.95 0.55 0

------------------------------------------------------------

Table S10. Taxonomic groups (Fig. 4C)

Group n x_bar_ y_bar_ inter slope r^2^ F_obs_ F_th_ P S

-------------------------------------------------------------

Bact 13 -14.93 2.59 -2.30 -0.33 0.07 0.84 4.84 0.38 0

BactNoPI 7 -14.87 2.30 1.02 -0.09 0.05 0.29 6.61 0.61 0

Inse 19 -4.43 1.95 2.36 0.09 0.14 2.82 4.45 0.11 0

Crus 19 -0.91 2.53 2.39 -0.15 0.03 0.55 4.45 0.47 0

Moll 5 -5.22 2.33 2.39 0.01 0.83 14.89 10.13 0.03 1

Fish 29 0.16 2.13 2.14 -0.06 0.04 1.17 4.21 0.29 0

Amph 31 -3.69 2.30 2.37 0.02 0.11 3.41 4.18 0.07 0

Rept 5 -1.03 2.16 1.97 -0.19 0.43 2.23 10.13 0.23 0

Bird 18 -0.62 1.94 2.05 0.18 0.31 7.31 4.49 0.02 1

Mamm 202 -0.78 2.20 2.20 7×10^-3^  0.01 2.32 3.89 0.13 0

-------------------------------------------------------------

Table S11. Taxonomic groups for locomotors (Fig. 4C)

Group n x_bar_  y_bar_ inter slope r^2^ F_obs_ F_th_ P S

----------------------------------------------------------------

Bact 10 -14.98 2.68 1.20 -0.10 7×10^-3^  0.06 5.32 0.82 0

Inse 17 -4.01 1.97 2.41 0.11 0.04 0.66 4.54 0.43 0

Crus 5 -0.43 2.14 1.95 -0.45 0.20 0.77 10.13 0.44 0

Fish 28 0.20 2.13 2.14 -0.06 0.05 1.27 4.23 0.27 0

Amph 25 -2.94 2.36 2.42 0.02 0.08 2.11 4.28 0.16 0

Rept 5 -1.03 2.16 1.97 -0.19 0.43 2.23 10.13 0.23 0

Bird 17 -0.67 1.92 2.04 0.17 0.29 6.18 4.54 0.03 1

Mamm 178 0.14 2.21 2.21 4×10^-3^ 2×10^-3^  0.39 3.89 0.53 0

----------------------------------------------------------------

Table S12. Functions (Fig. 4D)

Group n x_bar_  y_bar_ inter slope r^2^ F_obs_ F_th_ P S

-------------------------------------------------------------------

Non-loc 55 -6.23 2.26 2.40 0.02 0.10 6.03 4.02 0.02 1

Swim 53 -2.64 2.14 2.14 8×10^-4^  2×10^-4^  8×10^-3^  4.03 0.93 0

Fly 25 -2.61 1.85 2.11 0.10 0.16 4.39 4.28 0.05 1

Terr 216 -0.66 2.24 2.23 -0.02 0.06 14.81 3.89 2×10^-4^  1

TerrNoPI 210 -0.25 2.23 2.23 2×10^-3^  7×10^-4^  0.15 3.89 0.70 0

-------------------------------------------------------------------
